# Unveiling the topology of partially disordered micro-crystalline nitro-perylenediimide with X-aggregate stacking: an integrated approach†

**DOI:** 10.1039/d3sc05514k

**Published:** 2023-11-23

**Authors:** Renny Mathew, Aniruddha Mazumder, Praveen Kumar, Julie Matula, Sharmarke Mohamed, Petr Brazda, Mahesh Hariharan, Brijith Thomas

**Affiliations:** a Science Division, New York University Abu Dhabi P.O. Box 129188 Abu Dhabi United Arab Emirates brijiththomas@nyu.edu; b School of Chemistry, Indian Institute of Science Education and Research Thiruvananthapuram (IISER TVM) Maruthamala P.O., Vithura Thiruvananthapuram 695551 Kerala India mahesh@iisertvm.ac.in; c Department of Chemistry, Green Chemistry & Materials Modelling Laboratory, Khalifa University of Science and Technology P.O. Box 127788 Abu Dhabi United Arab Emirates sharmarke.mohamed@ku.ac.ae; d Advanced Materials Chemistry Center (AMCC), Khalifa University of Science and Technology P.O. Box 127788 Abu Dhabi United Arab Emirates; e Institute of Physics of the Czech Academy of Sciences Na Slovance 2/1999 18200 Prague 8 Czech Republic brazda@fzu.cz

## Abstract

Profound knowledge of the molecular structure and supramolecular organization of organic molecules is essential to understand their structure–property relationships. Herein we demonstrate the packing arrangement of partially disordered nitro-perylenediimide (NO_2_-PDI), revealing that the perylenediimide units exhibit an X-shaped packing pattern. The packing of NO_2_-PDI is derived using a complementary approach that utilises solid-state NMR (ssNMR) and 3D electron diffraction (3D ED) techniques. Perylenediimide (PDI) molecules are captivating due to their high luminescence efficiency and optoelectronic properties, which are related to supramolecular self-assembly. Increasing the alkyl chain length on the imide substituent poses a more significant challenge in crystallizing the resulting molecule. In addition to the alkyl tails, other functional groups, like the nitro group attached as a bay substituent, can also cause disorder. Such heterogeneity could lead to diffuse scattering, which then complicates the interpretation of diffraction experiment data, where perfect periodicity is expected. As a result, there is an unmet need to develop a methodology for solving the structures of difficult-to-crystallize materials. A synergistic approach is utilised in this manuscript to understand the packing arrangement of the disordered material NO_2_-PDI by making use of 3D ED, ssNMR and density functional theory calculations (DFT). The combination of these experimental and theoretical approaches provides great promise in enabling the structural investigation of novel materials with customized properties across various applications, which are, due to the internal disorder, very difficult to study by diffraction techniques. By effectively addressing these challenges, our methodology opens up new avenues for material characterization, thereby driving exciting advancements in the field.

## Abstract 2

An interdisciplinary approach to derive the structure of a disordered supramolecular system.

## Graphical abstract

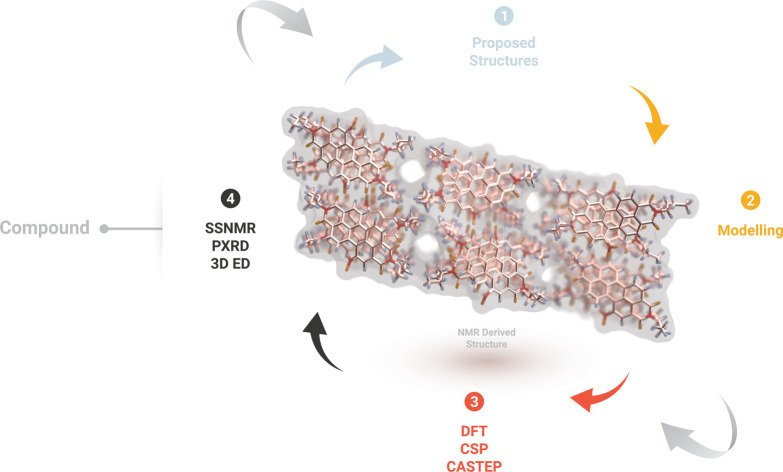

## Introduction

The precise understanding of the molecular structure and supramolecular arrangement of organic molecules is quintessential for the development of efficient photofunctional materials.^1–3^ Disorder in the crystal structure is a critical challenge to elucidate the atomic level arrangement of materials using conventional structure determination techniques. The efficacy of the single crystal X-ray diffraction (SC-XRD) technique is often limited when applied to materials exhibiting semi-crystalline and/or disordered properties.^4^ By definition, crystal structures contain different types of symmetries such as translational and point groups, which can be exploited to fully describe the arrangement of atoms in a material. Disordered structures exhibit a lack of or local deviation from the symmetries typically observed in well-ordered materials. As a result, traditional approaches for obtaining crystal structures or predicting packing using quantum mechanical modeling tools become challenging due to the presence of disorder.^5–8^

Perylenediimides (PDIs) are one of the most fascinating classes of dyes in the vast plethora of the rylene family.^9^PDIs are robust and have good photochemical and thermal stability, making them suitable candidates for multiple optoelectronic applications.^10–13^ There has been growing interest in understanding the structural packing in the supramolecular assembly of several PDI derivatives in recent years. In molecular solids, structural packing controls diverse significant physical phenomena, including charge transport, electron transfer, and electrical conductivity, among others.^14–17^ Sebastian *et al.* have recently published a study describing the phenomenon of null-exciton coupling in a perylene based chromophoric aggregate.^18^ The above work revealed the importance of crystal packing in the solid state for the effective electronic coupling responsible for the aggregate photophysical properties. Among PDIs, nitro-perylenediimide(s) (NO_2_-PDIs) are previously reported to exhibit light-induced excited-state photorearrangement,^19^ strong non-linear optical response,^20^ ultra-stable radical anion generation,^21^ and singlet fission in nanocrystal assembly.^22^ Furthermore, NO_2_-PDIs are considered efficient building blocks for developing functional organic materials. Therefore, understanding the supramolecular assembly and the nature of the crystal packing in NO_2_-PDI derivatives becomes vital for several potential applications.

PDIs without any substituents on the imide group crystallizes in the *P*2_1_/*n* space group and PDIs with two nitro groups on the bay position crystallize in the *Cc* space group (Fig. S1†) could be characterized using conventional SC-XRD techniques (Table S1†).^22^ The characterization of NO_2_-PDI by SC-XRD techniques is challenging due to a disorder causing substantial diffuse scattering, which significantly complicates the determination of the correct Laue class symmetry and accurate integration of the diffracted intensity.^23–25^ Characterizing molecular disorder on a macroscopic level is a difficult task. It may be accomplished through X-ray diffraction (XRD) techniques as well as various spectroscopic methods including solid-state NMR, Raman spectroscopy, and dielectric spectroscopy. Effectively discerning subtle variations in molecular disorder at the molecular level using single crystal X-ray or three-dimensional electron diffraction (3D ED) remains a hurdle, as it involves difficult analysis of the diffuse scattering, and thus the intensity diffracted between the Bragg spots.^23–26^ Additionally, the above-mentioned approach lacks a reliable way to measure the degree of disorder among the various crystals found in bulk powder. In contrast to diffraction approaches, which employ an average unit cell model, the use of solid-state NMR presents a method that analyzes short-range order in materials. The experiments involved span from microseconds to tens of milliseconds, during which radiofrequency pulse operations and time-domain signal acquisition take place. And it is possible to differentiate between static and dynamic disorder using solid-state NMR. When attempting to comprehend the variety of interactions present in a crystal lattice, a mixture of complimentary characterization procedures is usually to be advocated.

**Scheme 1:**
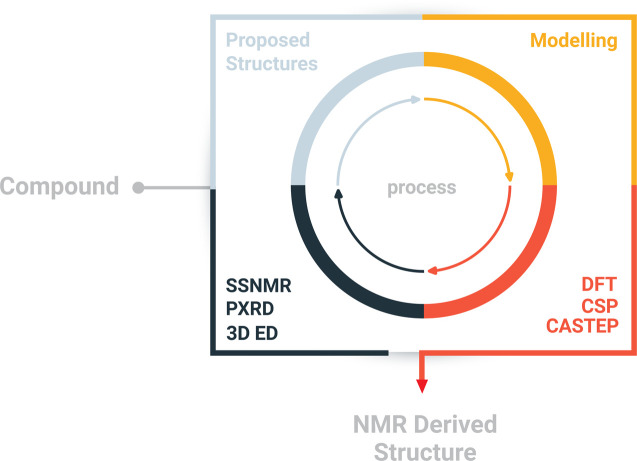
Schematic diagram of the structure determination approach. The approach combines complementary techniques like powder X-ray diffraction, electron diffraction, and solid-state NMR on a quantum mechanical platform to integrate the results.

Here, we report a multi-technique approach capable of elucidating the crystal structure of NO_2_-PDI. The multi-technique approach involves a combination of complementary techniques, including powder X-ray diffraction (PXRD), 3D ED, solid-state NMR and periodic density functional theory (DFT) calculations (Scheme 1 and Table S2†)^27^ for the structural characterization of materials on the atomic scale.^28–46^ The central premise of the approach is to integrate local structural information available from NMR with information from different approaches, particularly exploiting complementary diffraction techniques such as PXRD and 3D ED,^47^ which provide averaged information using the unit cell concept. The approach involves the following steps: (i) information from conventional chemical analysis techniques such as elemental analysis and solution NMR is used to determine the composition and chemical structure of the material. (ii) 3D ED experiments are used to obtain the unit cell parameters, symmetry of the average structure model and molecular packing including the conformation of alkyl chains and nitro groups. (iii) The solid-state NMR spectra of NO_2_-PDI are collected to identify the intermolecular correlation peaks to understand the spatial orientation of one molecule relative to another. Solid-state CPMAS (cross-polarization magic angle spinning) NMR is an ideal tool that provides an easier route to determine the molecular structure in disordered solids because the link between isotropic chemical shifts and bonding environments is specific to each atom. (iv) The structure model proposed by 3D ED is validated by comparing the findings from solid-state NMR about the mutual proximity of different parts of the NO_2_-PDI structure. Information about the mutual proximity is obtained from the intermolecular correlation peaks extracted from ^1^H–^1^H double quantum single quantum spectra and ^1^H{^13^C} HETCOR (cross polarization–heteronuclear correlation) solid-state NMR spectra. Molecular conformation, packing and unit cell parameters determined by 3D ED were further used for crystal structure prediction (CSP) and DFT calculations. Comparing the DFT predicted and observed NMR parameters and PXRD patterns provides a final check on the structure. The proposed crystal packing is in the *Pbca* space group with the perylene core stacked in an X-shaped packing fashion and contains disordered alkyl tail groups. Relatively few methods are available to deal with the packing of moderately sized organic molecules with intrinsic disorder during supramolecular self-assembly.^31,40,48–50^ A complementary approach was employed by Smalley *et al.* to unravel the structure of the β polymorph of l-tyrosine.^51^

## Results and discussion

**Fig. 1:**
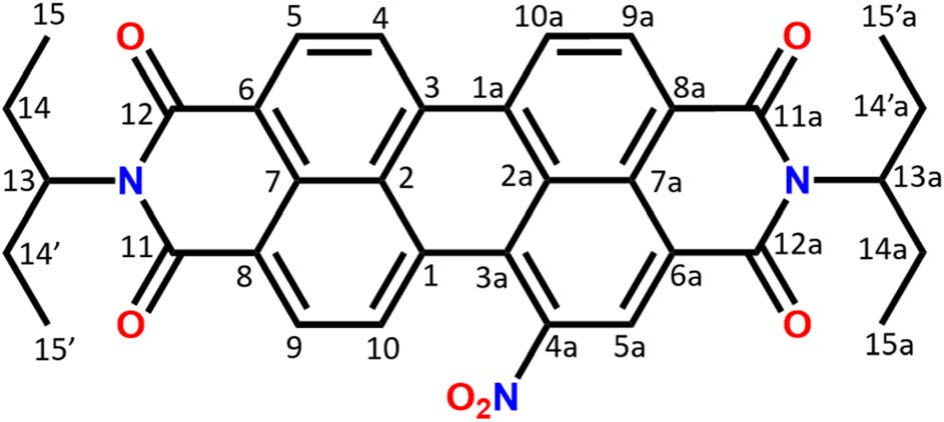
Structure of NO_2_-PDI with the numbering scheme used in the article. The system consists of an aromatic moiety and alkyl tails with distinct carbon and proton chemical shifts. The aromatic ring makes the core region planar with the possibility of π–π stacking, whereas the alkyl tail protrudes in upward and downward directions.

The composition and chemical structure of the NO_2_-PDI molecule were determined using elemental analysis and solution-state NMR. The molecular formula was determined to be C_34_H_29_N_3_O_6_ by elemental analysis. The measured density is around 1.3 g cm^−3^ (see the ESI†), which is consistent with the density of similar perylene compounds in the literature (Table S3†).^52–59^ The solution NMR collected in CDCl_3_ solvent confirms the NO_2_-PDI monomer structure illustrated in Fig. 1. The solution-state NMR chemical shift of the monomer is assigned with the help of ^1^H, ^1^H–^1^H COSY (correlation spectroscopy), ^1^H–^13^C HSQC (heteronuclear single quantum correlation), and ^13^C one dimensional nuclear magnetic resonance spectra (see the ESI (Fig. S2–S4)†).

### 3D electron diffraction

**Fig. 2:**
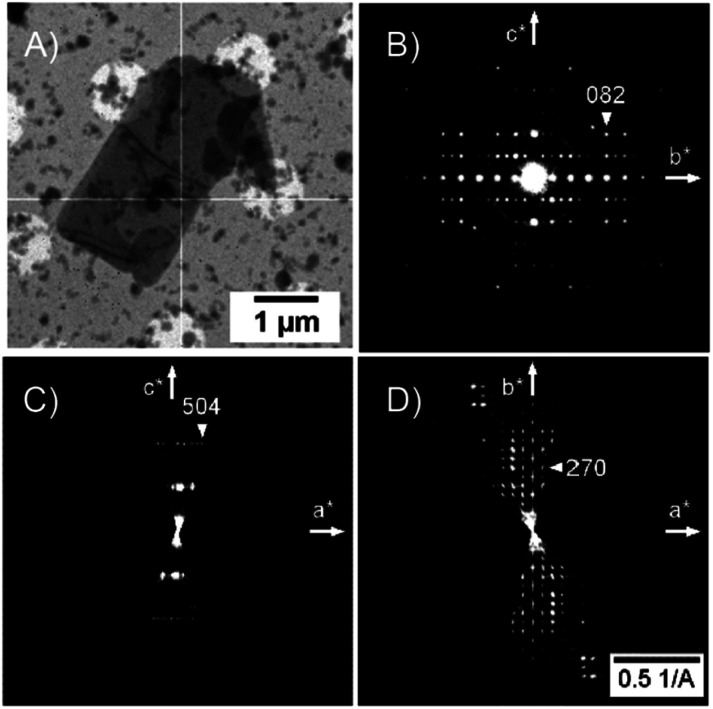
(A) The TEM image of the crystal of NO_2_-PDI. Tiny crystallites are ice crystals formed during cryo-transfer of the samples into the microscope. Bend contours are apparent in the middle and in the lower part of the crystal. (B–D) Reciprocal space sections of NO_2_-PDI (0*kl*, *h*0*l* and *hk*0). Violations of the systematic absences observable in the 0*kl* section (*b*-glide plane) and in the *hk*0 section (*a*-glide plane). These violations are less sharp and smeared along the a* direction indicating that they relate to structure disorder.

The 3D electron diffraction method was used because the crystals were too thin platelets for X-ray crystallography. Thanks to the much greater interaction of electrons with matter it was possible to obtain diffraction data. The NO_2_-PDI material was rather stable in the electron beam. The crystals adopt a shape of thin platelets with the [100] direction perpendicular to the platelet. A total of 23 crystals were subjected to measurement. Diffraction data suffered from diffuse scattering, which was present to varying degrees in all datasets. The diffuse streaks run along the [100] direction. The platelets were rather large (lateral dimensions were usually below 10 microns) and bent (Fig. 2), which amplified the smearing of the intensities along the [100] direction. Three data sets had an acceptable level of diffuse scattering and crystal mosaicity. Fig. 2 shows the reciprocal space sections of one of them indicating that the space group symmetry is very probably *Pbca* but at the same time, the systematic absences are violated to a relatively large resolution. This decreases the probability that these violations are caused by multiple scattering and thus that they are a result of violation of the *Pbca* symmetry. Moreover, these violating diffraction maxima are smeared along the [100] direction, which connects them with the internal disorder along this direction.

**Fig. 3:**
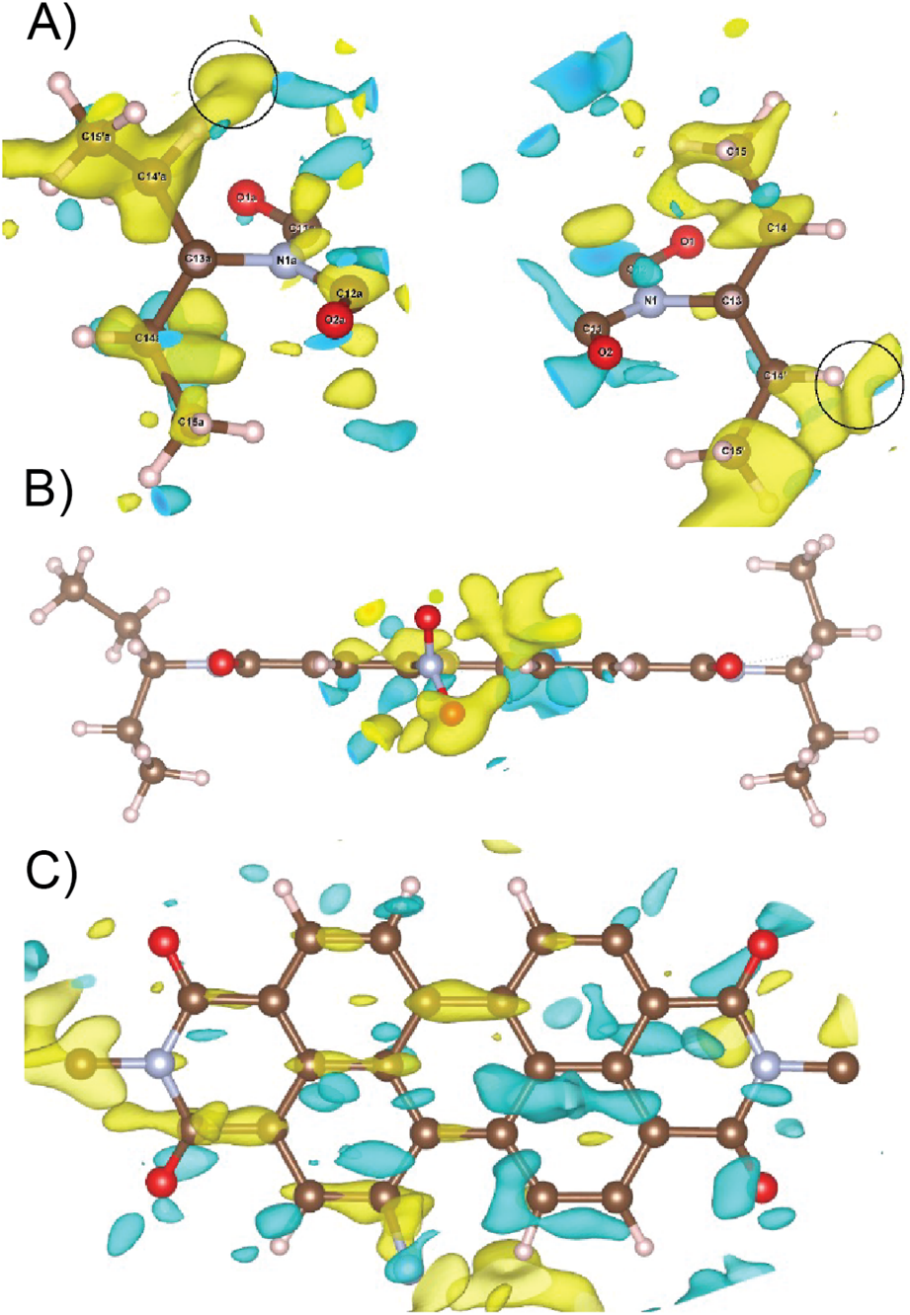
(A) Difference potential map after refinement of the model without side chains, focused on the volume where these side chains are expected (2 sigma level, yellow positive, and cyan negative). This difference potential was interpreted by inserting a model molecule with correct bond lengths and angles and adjusting its conformation. The C15′a methyl group is rotated out from the core of the molecule. Black circles mark the position of minor positive difference potential maxima indicating possible alternative orientations of the methyl groups C15′a and C15′. (B) Difference potential at the 2 sigma level around the nitro group (yellow positive and cyan negative) after refinement of the model without side chains and with the nitrogen atom of the nitro group. (C) Difference potential at the 2 sigma level (yellow positive and cyan negative) around the core part of the molecule.

Data reduction was done using PETS2 and the calibrated optical distortions were applied to obtain as accurate lattice parameters as allowed by the diffuse scattering (*a* = 36.1(6) Å, *b* = 19.23(7) Å, *c* = 7.97(5) Å, *α* = 90°, *β* = 90°, and *γ* = 90°). The structure was solved in *Pbca* using Superflip.^60–62^ All atoms were found in the solution (Fig. S5†). The molecular arrangement corresponded to the X-shaped packing with a rotation angle of about 46° between the adjacent perylene molecules. The bond lengths and angles between atoms of the aromatic core part of the molecule corresponded well to the perylenediimide geometry. However, some of the bond lengths and angles within the alkyl sidechains and the nitro group were relatively far from the expected values. Therefore, the atoms of these groups were removed from the structure model and the refinement against data combined from three best crystals used only the core part of the molecule. The purpose of this step was to test if the data contain sufficiently strong information about the side chains of the molecule. After kinematical refinement (refinement omitting multiple scattering due to the strong interaction of electrons with matter) in Jana2020, there was not clear information about the side chains in the difference potential map (an analogue to the difference Fourier map in X-ray crystallography).^63^ Therefore, it was necessary to model the multiple scattering using dynamical refinement.^64,65^ After the refinement, it was possible to localize the positive maxima due to the atoms of the aliphatic tails (Fig. 3). It was possible to interpret the difference potential only with the help of the MCE program by inserting the molecule model into the difference potential and adjusting the torsion angles.^66^ The maxima were rather low and broad indicating significant disorder within this part of the molecule. This was confirmed after the refinement of the whole molecule, where the atomic displacement parameters (ADPs) of the tails were more than three times larger than those of the core of the molecule. The dihedral angles C11–N1–C13–H13 and C12a–N1a–C13a–H13a were 17° and 12°, respectively, well away from the most energetically favourable 0° for a molecule in vacuum. When the molecule is viewed from the top, both H13 and H13a point to the same side of the molecule (Fig. 3). Moreover, the C15′a methyl group was rotated out from the core part probably due to steric demands imposed by the neighboring molecule. A significant positive difference potential was observed next to the nitrogen atom, where the nitro group was found in the structure model obtained from the phase problem solution. However, the difference potential did not clearly indicate the position of one of the oxygen atoms in the nitro group. However, another large cloud of positive maxima was observed next to atom C10, which might be interpreted as an alternative position of the nitro group within the average model structure (Fig. 3).

Diffuse scattering in the data and broad maxima in the difference potential indicate significant disorder in the material. One of the sources of this disorder is probably the nitro group, because in the average structure, the nitro groups of the two neighbouring molecules are too close together (just over 2 Å). This unexpectedly short distance is probably compensated for by the slight shift of the molecules along their long axis (Fig. S5 and S6†). This situation would correspond to the observed elongated difference potential in the direction of the long axis of the molecule around the atoms of the core of the molecule (Fig. 3). Furthermore, it is possible to observe minor maxima in the difference potential in Fig. 3A (black circles), which may indicate the alternative conformations of the alkyl chains, namely the orientations of the methyl groups C15′a and C15′. These features indicate that sliding of the molecules along the long axis causes rearrangement of these methyl groups to conform with the neighboring molecules. The disorder of the nitro group, suggested by the cloud of the positive difference potential around the C10 atom, may be interpreted in two ways. First, the orientation of the molecule (and thus the orientation of the nitro group) is disordered within the columns of the π-stacked molecules, and thus along the [001] direction. This possibility is less probable, because there is not enough room in the average structure to allow this. Second, the more probable type of disorder is the flip of the molecule orientation in the adjacent columns, when we move along the [100] direction. The interactions in this direction between the adjacent columns of the π-stacked molecules are mediated only by the region of the alkyl chains. This interaction may not be strong enough to carry the information about the position of the nitro group in the adjacent column. This would be consistent with the observation of the diffuse streaks along the same direction and strong violation of the *a*-glide plane of the *Pbca* space group, which connects the molecules within the unit cell by symmetry along the [100] direction.

The dynamical refinement (Table 1) converged to *R*_obs_ = 22.3%, which is significantly higher than the usual *R*-factor observed for this type of refinement of the 3D ED data (5–15%). The above-mentioned fact indicates that the description of the material using an average unit cell with *Pbca* symmetry is insufficient and the introduction of a disorder would probably improve the *R*-factors. However, the difference potential features are so broad and shallow, and it also contains relatively high noise that they can be easily misinterpreted.

**Table 1:** Crystallographic details of 3D ED dynamical refinement against combined data from three crystals

Stoichiometric formula	C_34_H_29_N_3_O_6_
Molecular weight	575.6
Temperature, K	100
Crystal symmetry	Orthorhombic
Space group	*Pbca*
*a*, *b*, *c* (Å)	36.1(6), 19.23(7), 7.97(5)
*Z*	8
Number of frames	32 + 13 + 32
Rotation semi-angle (°)	0.3
Resolution (Å^−1^)	1.0
*g*_max_, *S*^max^_g_ (matrix), *S*^max^_g_ (refine), *R*_Sg_, *D*_Sg_	1.0, 0.01, 0.1, 0.66, 0.001
Number of meas./obs. refl. (*I* ≥ 3*σ*(*I*))	15 577, 4293
Number of refined parameters	304
*R*_obs_ (%), w*R*_all_ (%), GoF_obs_	22.29, 22.89, 7.31
Thickness (nm, refined)	129(1); 241(1); 144(1)

### Solid-state NMR

**Fig. 4:**
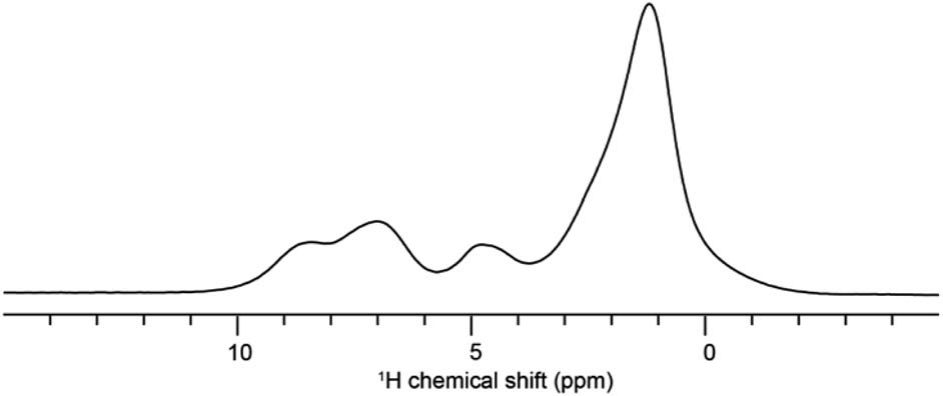
The ^1^H spectra of NO_2_-PDI collected at room temperature with 100 kHz spinning.

**Fig. 5:**
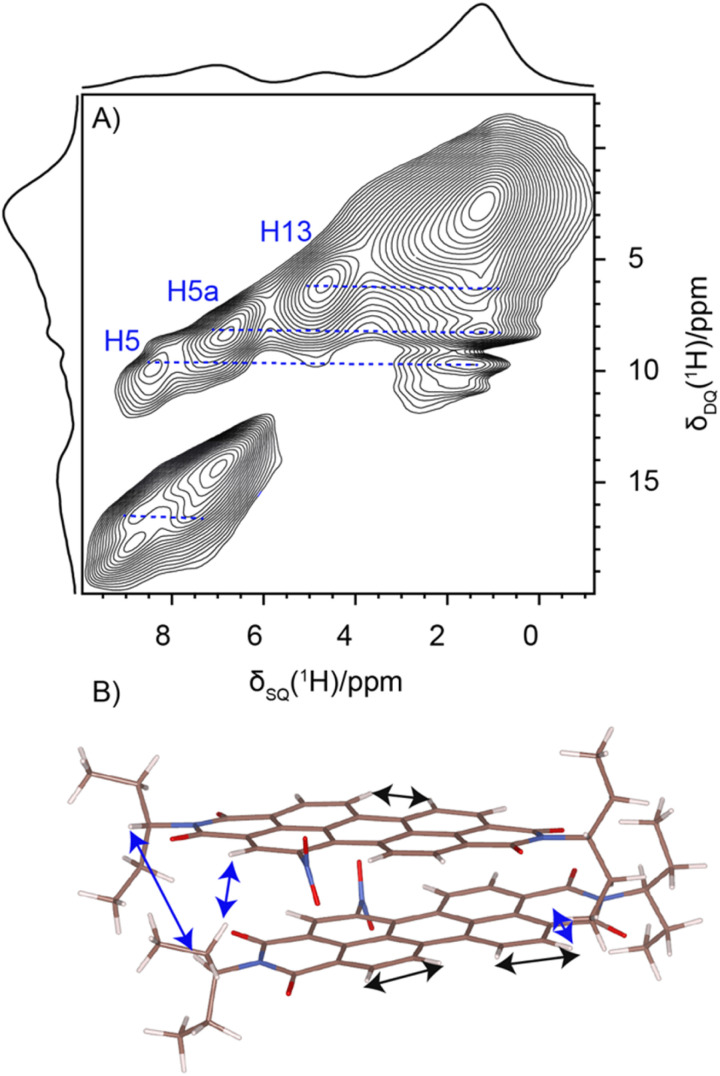
(A) ^1^H–^1^H DQ–SQ NMR spectrum of NO_2_-PDI collected at 60 kHz spinning speed at room temperature, using the BABA pulse sequence with a recoupling time of two rotor periods corresponding to 33 μs. (B) Corresponding correlation is also shown.

**Fig. 6:**
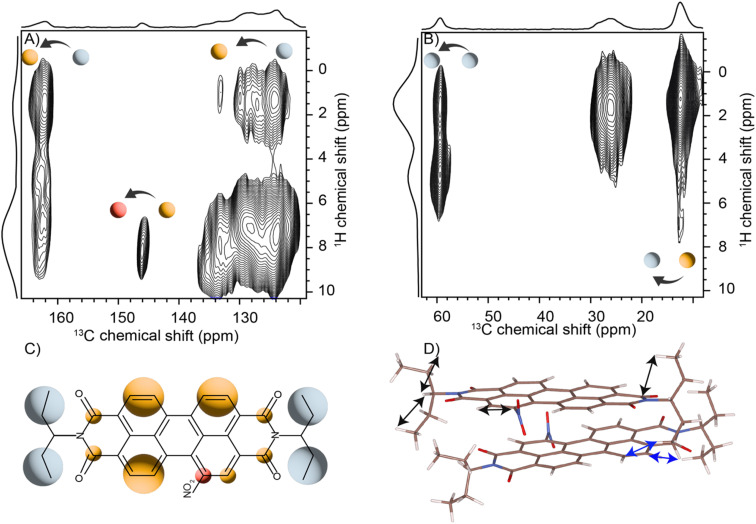
(A) The two dimensional ^1^H{^13^C} CP-HETCOR solid-state spectra of the NO_2_-PDI sample obtained at 1 ms: (A) aromatic and (B) aliphatic regions. The ^1^H sum projections corresponding to aliphatic and aromatic regions are also shown. The spectra were collected at 60 kHz spinning speed at room temperature. The folding of alkyl tails along the aromatic carbon is observed from the ^1^H{^13^C} correlation peaks. (C) Structure of the molecule with the colour shade in line with the correlation shown in (A) and (B). (D) The arrows showing inter- (blue) and intra- (black) molecular interaction observed in the HETCOR in the proposed model.

Solid-state NMR experiments were carried out to ascertain the connection between the NO_2_-PDI monomer and crystal packing. The ^1^H spectrum of NO_2_-PDI collected at room temperature with 100 kHz spinning speed is shown in Fig. 4. The *T*_1_ of aliphatic protons is 1.7 s, whereas the *T*_1_ of aromatic proton is 2.3 s (Fig. S7†). The correlations observed in ^1^H–^1^H DQ–SQ (double quantum single quantum) MAS arise from the interactions between pairs of hydrogen nuclei which are close by in space in NO_2_-PDI. In the ^1^H–^1^H DQ–SQ MAS NMR spectrum of NO_2_-PDI, a pronounced self-correlation is evident, originating from the aliphatic protons located closely together within the alkyl tails. Fig. 5 (Fig. S8–S10†) shows the correlation between the alkyl proton and aromatic proton and this points toward a proximity of the proton in the alkyl tail and the aromatic proton, which is possible only in the staggered stacking arrangement as shown in Fig. 5B. No correlation is observed between 9a and the alkyl tail, which means that the X-shaped packing is possible only in one way. The correlation between H13 and CH_3_–CH_2_– is intramolecular, whereas the correlation between H5a and H5 is intermolecular. Furthermore, if the structure is stacked on top of each other, detecting the above-mentioned correlation becomes challenging. In the aromatic region, proton signals exhibit overlapping, indicating a combination of self-correlation peaks and mostly intramolecular correlation peaks. Interestingly most of these correlations shown in Fig. S8† are obtained at a short recoupling time of 16 μs, which also indicates the proximity of these groups. Longer durations of dipolar coupling will probe further intermolecular distances as the build-up of dipolar coherence is dependent on the magnitude of the dipolar coupling constant between two ^1^H spins. To obtain further structural constraints, a 2D ^1^H{^13^C} heteronuclear (HETCOR) experiment was performed (Fig. 6 and S11–S13†). And the above-mentioned experiment correlated the ^1^H–^13^C spin pairs in close spatial proximity. In line with ^1^H–^1^H DQ–SQ spectra, correlations are observed in ^1^H{^13^C} HETCOR between the proton on the aliphatic tail and carbons on the aromatic core. A correlation between aromatic proton and aliphatic carbon is also observed, which is in line with the above observation (Fig. 6B). In the ^13^C aliphatic region, two distinct types of new correlation peaks are observed in the long contact time compared with short contact time. One originates from the protons in the aromatic group to the CH_2_ and CH_3_ groups, primarily signifying intermolecular interactions. And the other correlation peak is formed from the nearby methyl group transferring polarisation to C13 and C13a carbon. In the ^13^C aromatic region, correlation between the aliphatic tail and carbonyl group is observed. The CH_3_ shows correlation with carbonyl, and it could be either inter- or intra-molecular. The correlation peak shown in the ^1^H{^13^C} HETCOR spectra should be intramolecular from the nearby protons on the C–NO_2_ carbon atom. Correlation between the aromatic proton and carbonyl group is also observed in the ^1^H–^13^C HETCOR at long mixing time. Relatively sharp peaks in the solid-state NMR spectra point towards periodic packing in the sample. In the ^1^H{^13^C} HETCOR correlation spectra collected at a long mixing time of 1 ms (Fig. 6, S11 and S12†), intermolecular correlation peaks are observed, which gives more information about the spatial orientation of one molecule with respect to another (Table 2). The intermolecular correlations in the system can be classified into the following categories: (a) from the alkyl tail to aromatic –CH carbons, (b) from the alkyl tail to the CO group, which is primarily intramolecular, (c) from the aromatic –CH carbon to the alkyl tail and (d) from the aromatic –CH carbon to the quaternary carbon on the aromatic ring. In the above-mentioned correlation peaks, (a) and (c) are intermolecular and point towards a packing in which perylene stacks in a columnar fashion with tails projecting in between (Fig. 6).

**Table 2:** Set of correlation peaks obtained from ^1^H{^13^C} CP-HETCOR spectra

Numbering of the proton attached to the carbon atom	Numbering of carbon atoms	Category of correlations
9a, 10a, 4, 5, 9, 10, 5a	15, 14, 15′, 14′, 15′a, 14′1, 15a, 14a	Intermolecular
15, 14, 15′, 14′, 15′a, 14′1, 15a, 14a	11, 12, 11a, 12a	Intramolecular
15, 14, 15′, 14′, 15′a, 14′1, 15a, 14a	9a, 10a, 4, 5, 9, 10, 5a	Intermolecular
9a, 10a, 4, 5, 9, 10, 5a	7a, 7	Intermolecular

### Interface of solid-state NMR and 3D ED

The structure determination of NO_2_-PDI is challenging due to the disorder present in the system. The alkyl tail on the imide substituent introduces disorder into the PDI derivatives and makes it challenging to characterize by conventional methods. This is consistent with molecular mechanics calculations, which indicate that most of the conformational variability in NO_2_-PDI arises as a consequence of different orientations in the alkyl tails (Fig. S14†). Here we used the correlation and chemical shift information from solid-state NMR to address the above-mentioned challenge. The correlation data provide insights into how perylenediimide molecules are oriented in relation to each other. The possible correlation from the two-dimensional NMR is shown in Fig. 5 and 6. The chemical shift can be employed to assess and compare the various models constructed based on the experimental data. To address the potential disorder in the orientation of molecules within the stacked column, we explored four distinct scenarios, as illustrated in Fig. S15.† (i) Case 1 involves the structure suggested by 3D ED analysis. (ii) In case 2, the structure includes the rotation of the alkyl tail. (iii) In case 3, the structure features the flipping of the NO_2_-PDI molecule along its long axis. (iv) Case 4 entails the structure with the NO_2_-PDI molecule flipped along its short axis. The refinement of the models was subsequently carried out by utilizing correlation information acquired from solid-state NMR and by simulating chemical shifts in solid-state NMR (Table 3). The first case uses the average structure model derived from the 3D electron diffraction data. The obtained unit cell was optimized for unit cell parameters, but no significant variations in the parameters were observed *a* = 36.43 Å, *b* = 19.33 Å, *c* = 7.85 Å, and *α* = *β* = *γ* = 90°. The NMR chemical shift values are also simulated on the optimised cell, and no improbable change was observed (Table S4†). The calculated ^1^H NMR chemical shift and the measured solution-state NMR chemical shift are shown in Table S5† for reference. Structural models for the remaining three scenarios were constructed to consider the disorder and various possible orientations within one molecular column, which will be discussed in the subsequent section. There is a probability of different cases existing together with case 1 as the predominant species. With the current resolution it is difficult to quantify the ratio and the other cases are present in a very low amount.

**Table 3:** The root mean square deviation of proton chemical shift and relative energy of four different models to address the disorder

S. no.	Cases	RMSD ^1^H (between experiment and CASTEP)	Relative energy (kcal mol^−1^)
1	Case 1	0.49 ppm	0
2	Case 2	0.89 ppm	22.76
3	Case 3	1.20 ppm	12.69
4	Case 4	1.03 ppm	−2.6

### Elucidating the X-shaped packing topology of NO_2_-PDI

The aromatic planar perylene core tends to stack in an X-shaped packing with a rotation angle (*α*) of 45.72° between the adjacent perylene molecules in the columnar stack (Fig. S15†). The stacking of the NO_2_-PDI molecule in an X-shaped packing arrangement is analogous to the Br derivative of PTE (perylene-3,4,9,10-tetracarboxylic tetra-alkyl esters).^67^ The dipole moment in the NO_2_-PDI monomer amounts to 5.26 debye. The X-shaped stacking is driven by the π–π stacking and antiparallel alignment of dipole moment.^68^

As explained before, specific correlation between the alkyl tail and aromatic region close to the nitro group indicates X-aggregate stacking. In the average model derived from 3D ED, the distance between the oxygen atoms of the neighbouring nitro groups is only about 2 Å as shown in Fig. S8.† To further understand the same, we did CASTEP calculation on a system flipped along the short axis and the long axis (cases 2 and 3) (Fig. S15†). The RMSD in the ^1^H chemical shift is around 1.03 ppm and 1.20 ppm respectively, which is more than the acceptable value (Table S6†). In short the RMSD in the ^1^H NMR chemical shift in case 2 and case 3 helped to eliminate the possibility of the structure to have the NO_2_ group to switch its position within one column of the molecules.^23^ In order to determine the oxygen position within the nitro group, it is necessary to conduct additional solid-state NMR experiments utilizing ^17^O labelling. However, this task presents challenges due to the nitro group's paramagnetic nature, as indicated by the findings reported in electron paramagnetic resonance (EPR) studies. DFT calculations also point towards the fact that the predicted X-shaped packing is relatively stable compared to the other orientations of perylene stacking (Fig. S17†). The stacking of one molecule above another with zero angle between the planes of two molecules results in steric hindrance between the tails, which is considered geometrically unfavourable.^68,69^ The alkyl tail also contributes to disorder, in addition to the NO_2_ group at the bay substituent, which will be discussed in the following sections.

The proximity of the methyl group relative to the aromatic proton is obtained from the ^1^H–^1^H DQ–SQ spectra. There is a distribution of isotropic chemical shift in the solid-state NMR spectra, which points towards the disorder. The distribution of isotropic chemical shift observed in the methyl group and ethyl group is evident from the proton and carbon dimension in ^1^H{^13^C} HETCOR and ^1^H–^1^H DQ–SQ spectra, which is observed in the short recoupling time also. In the 3D ED studies (Fig. 3), the C15′a is oriented in the outward direction relative to the other methyl groups. In the ^1^H–^13^C HETCOR, the correlation between the methyl group and carbonyl group is observed, which could be either inter- or intra-molecular. To address this, we used case 4, in which the methyl group is rotated inward to see the difference (Fig. S15†). The RMSD of ^1^H chemical shift between experiment and calculated is around 0.89 ppm and the model has a relatively higher energy compared to case 1.

### Crystal architecture

**Fig. 7:**
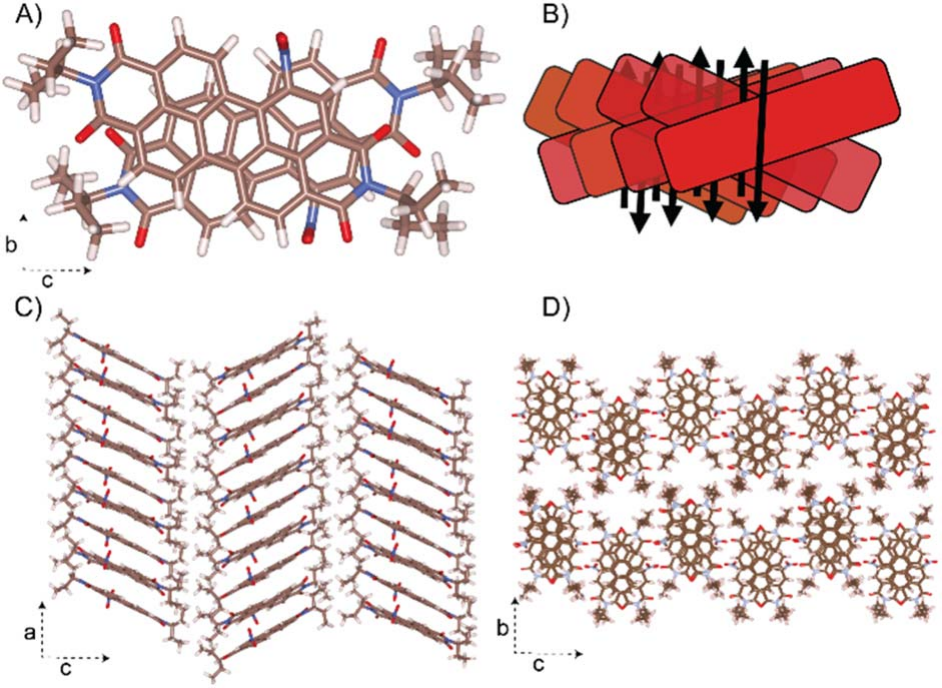
Proposed crystal structure of NO_2_-PDI. The unit cell parameters are *a* = 36.1 Å, *b* = 19.23 Å, *c* = 7.97 Å, and *α* = *β* = *γ* = 90°. (A) The aggregation of NO_2_-PDI is shown for a dimer. (B) Schematic drawing of the overlap in an X-aggregate fashion is also shown. The dipole moments (black arrow) are in the antiparallel orientation in each columnar stack and thus minimised. (C) The columnar stacking of the NO_2_-PDI monomer along the ‘*a*’ axis is shown. (D) The stacking of NO_2_-PDI molecules onto the ‘*bc*’ plane.

Remarkably, the NO_2_-PDI molecules exhibit a unique stacking mode, where the long axis of the adjacent molecules is packed in a cross at the 1D column (Fig. 7). One of the NO_2_-PDI molecules is rotated by 45.72° with respect to the other, and the two nearby molecules have overlapping aromatic core perylene rings. The strong π–π interaction existing in the adjacent perylene core leads to an inter-layer distance of around 3.5 Å. The perylene cores are stacked one above the other, with tails orienting upward and downward with respect to the plane containing the perylene core. The steric hindrance between the alkyl tail on the imide substituent drives the assembly into the antiparallel dipole stacking instead of the paralleled construction.^68^

Symmetry-adapted perturbation theory-based energy decomposition analysis (SAPT-EDA) was performed to gain insights into the stability of the intermolecular interactions in the X-dimer in NO_2_-PDI. SAPT is an advanced computational tool, which directly calculates the energies of non-covalent interactions between two molecules without calculating the total energy of the NO_2_-PDI monomers.^69^ The X-dimer exhibited a total interaction energy of −160.81 kJ mol^−1^, where dispersion was found to be the significant stabilizing component with an energy value of −211.94 kJ mol^−1^ (Table S9†). The significant negative total pairwise interaction energy for the X-dimer verified the stabilizing nature of the intermolecular interactions in the solid-state packing of NO_2_-PDI.

Photoluminescence experiments were carried out to corroborate the nature of X-dimers in the solid-state packing of NO_2_-PDI. The solid-state excitation spectra show a broad featureless, red-shifted peak characteristic of an X-shaped packing compared to the NO_2_-PDI monomer excitation peak (Fig. S18 and Table S10†).^67,70^ The emission spectra of NO_2_-PDI in the solid-state display an enhanced, red-shifted peak in comparison to the monomer emission spectra. The observed solid-state photophysical properties of NO_2_-PDI are in accordance with the spectral features of a previously reported X-shaped packing architecture of a perylene derivative.^67^

The electrostatic surface potential (ESP) map of NO_2_-PDI was computed to explain the critical role played by the electrostatic component in dictating the solid-state packing of NO_2_-PDI.^71,72^NO_2_-PDI displays an intermediate positive potential at the aromatic core (Fig. S18†).^6^ The nitro and carbonyl groups display a strong negative potential due to the presence of oxygen lone pairs (Fig. S18†). The synergistic electronic effects of nitro and carbonyl groups make the bay C–H bonds electrophilic and therefore, the bay C–H bonds opposite to the nitro group display an intermediate to strong positive potential (Fig. S19†). The X-type dimer formation in the unit cell and throughout the solid-state packing can be rationalized from the electrostatic attractive interactions stabilizing the dimer. The dipole moment cancellation (Table S8†) from the NO_2_-PDI monomer (5.27 D) to the dimer (3.59 D) explains the favorability of forming an X-type dimer in the solid-state packing of NO_2_-PDI. The presence of two nitro groups in the bay position results in molecular symmetry, favoring J-aggregate stacking rather than X-aggregate stacking.^22^ In a recent study, Sebastian *et al.* reported an akin trend in the case of a Br-substituted PDI derivative.^67^ The PXRD pattern of NO_2_-PDI is collected (Fig. S20†) and compared with the simulated PXRD pattern. A close match is observed between the experimental and simulated powder XRD patterns. The mismatch in intensity at a higher angle might be due to the disorder arising from the alkyl tail and nitro group. The NMR parameters were simulated using CASTEP, as depicted in Fig. S20 and detailed in Tables S4, S5 and S10.† The results showed a favorable agreement between the simulated and observed NMR parameters.

## Conclusions

In conclusion, the supramolecular architecture of moderately sized functional organic molecules with inherent heterogeneity is deduced through the sophisticated integration of solid-state NMR and 3D electron diffraction. The elusive crystal packing of NO_2_-PDI with disordered tails and nitro functional groups was determined by utilising the aforementioned methodology. Solid-state NMR provides information over a short range of a few nano-meters to give insights into the material's local structure, chemical environment, and spatial arrangement. Meanwhile electron diffraction delivers information over a long-range arrangement providing information about the average structure represented by the unit cell. The obtained structure is validated *via* DFT calculations that allow us to simulate NMR parameters. The derived structure of NO_2_-PDI has an average *Pbca* symmetry with the stacking of the perylene core in an X-shaped packing fashion and this average structure is strongly disordered including all parts of the molecule: alkyl tails, the nitro group and the core. Disorder results from the flexibility and ability of alkyl tails to adopt a variety of conformations in the crystal lattice. The cross-transition dipole structure of the perylenediimide moiety indicates that NO_2_-PDI will have small energy splitting and will allow all the optically excited states. To understand the supramolecular structure of similar systems where conventional approaches have difficulty, it is currently necessary to combine complementary techniques. We anticipate that further progress in employing complementary techniques will facilitate the exploration of structure–property relationships and, consequently, enable the discovery of novel functional organic materials.

## Data availability

All experimental/computational data and procedures are available in the ESI.† The crystallographic information file was deposited in the CSD database (No. 2306803).

## Author contributions

All authors have given approval to the final version of the manuscript.

## Conflicts of interest

There are no conflicts to declare.

## Supplementary Material

SC-015-D3SC05514K-s001

SC-015-D3SC05514K-s002
