# Supplementary material for: Unveiling the topology of partially disordered micro-crystalline nitro-perylenediimide with X-aggregate stacking: an integrated approach
Source: Chem Sci. 2023 Nov 23;15(2):490–9. doi: 10.1039/d3sc05514k (PMC10762722; doi:10.1039/d3sc05514k)
Supplement: SC-015-D3SC05514K-s001 [file SC-015-D3SC05514K-s001.pdf]

Electronic Supplementary Material (ESI) for Chemical Science.
This journal is © The Royal Society of Chemistry 2023

Unveiling the topology of partially disordered micro-crystalline nitro-perylenediimide with X-aggregate stacking: an integrated approach

Renny Mathew[a‡], Aniruddha Mazumder[b‡], Praveen Kumar[a], Julie Matula[a], Sharmarke Mohamed[cd]*, Petr Brazda[e]*, Mahesh Hariharan[b]* and Brijith Thomas[a]*

[a]Science Division, New York University Abu Dhabi, P.O. Box 129188, Abu Dhabi, United Arab Emirates;

[b]School of Chemistry, Indian Institute of Science Education Research Thiruvananthapuram (IISER TVM), Maruthamala P.O., Vithura, Thiruvanathapuram 695551, Kerala, India;

[c]Department of Chemistry, Green Chemistry & Materials Modelling Laboratory, Khalifa University of Science and Technology, P.O. Box 127788, Abu Dhabi, United Arab Emirates.

[d]Advanced Materials Chemistry Center (AMCC), Khalifa University of Science and Technology, P.O. Box 127788, Abu Dhabi, United Arab Emirates.

[e]Institute of Physics of the Czech Academy of Sciences, Na Slovance 1999/2, 18200 Prague 8, Czech Republic;

Table of Contents

Section 1: Materials and Methods……………………………………………...SI 5

1.1 Materials………………………………………..................................SI 5

1.2 3D Electron diffraction ……………………………………………...SI 5

1.3 Solution-state NMR ………………………………………………….SI 5

1.4 Solid-state NMR………………………………………………….….SI 6

1.5 DFT calculation…………………………….. …………………….SI 7

1.6 Symmetry Adapted Perturbation Theory (SAPT) Analysis ……………....SI 8

1.7 Photoluminescence Measurements ………………………………...SI 8

1.8 PXRD ………………………………………………………………….SI 8

1.9 Density Measurement………………………………………..……….SI 8

1.10 Conformer Distribution Search ………………………….....................SI 8

Section 2: Tables…………………………………………………………….SI 10

Table S1: Packing of related molecule from the literature……………………....SI 10

Table S2: Summary of the information ……………………..………………..SI 10

Table S3: Density of the related compounds from the literature……….............SI 11

Table S4: Experimental and calculated ^{13}C -NMR chemical shift of the **NO_2-PDI** molecule. ……………………………………………………..…………....SI 12

Table S5: The ^{1}H-NMR chemical shifts of **NO_2-PDI**…………………………...SI 13

Table S6: Dipole moment of the monomer and X-dimer of **NO_2-PDI** computed at cam-b3lyp/6-311+g(d,p) level of theory……………………………………......SI 14

Table S7: The experimental chemical shift of ^{1}H and calculated chemical shift of four different cases ……………………………………………………………...SI 15

Table S8: Total intermolecular interaction (SAPT(0)) energies and its components for X-dimer……………………………………………………………………....SI 15

Table S9: Excitation and emission properties of **NO_2-PDI** in solution-state (monomer) and solid-state (aggregate)……………………………………...…..SI 16

Table S10: Solution and solid-state ^{13}C-NMR chemical shift assignments of the **NO_2-PDI** molecule…………………………………….............................SI 16

Section 3: Figures………………………………………………………………SI 17

Figure S1: The structure of **NO_2-PDI** and related molecules……………………SI 17

Figure S2: ^{1}H-NMR spectra of the sample collected at room temperature on 500 MHz instrument……………………………………………………………………….SI 17

Figure S3: ^{1}H-^{1}H COSY spectrum of the sample collected at room temperature on 500 MHz instrument…………………………………………………………….SI 18

Figure S4: The ^{1}H-^{13}C HSQC spectra of the sample showing all the CH carbon atoms………………………………………………………………………………SI 18

Figure S5: Solution of **NO_2-PDI** (A), optimized molecule in Molview inserted into the place of the solved molecule position (B), overlay of the two structures (C, optimized molecule with one third of the intensity of the solution)…………………………………………………………………...SI 19

Figure S6: Difference potential at 2 sigma level after constrained core refinement with anisotropic ADPs with inserted MolView optimized molecule to best represent the difference potential features in the peripheral parts of the molecule (A). Difference potential at 2.5 sigma level after the whole molecule constrained refinement (B). The distance between the oxygen atoms of the neighboring nitro groups (dashed line) is only about 2.0 Å. The absolute value of the isosurface level for both parts of the figure is 0.40 e.Å-1. The unit cells are viewed along [100] direction.………………....SI 20

Figure S7: The T1 measurement with monoexponential fit is shown here. The T1 of aliphatic tails are short indicating the mobility or disorder of the species at room temperature.……………………………………………………………….…SI 21

Figure S8: ^{1}H-^{1}H DQ-SQ NMR spectrum of **NO_2-PDI** collected at 60 kHz spinning speed at room temperature, using the BABA pulse sequence with a recoupling time of one rotor periods corresponding to 16 μs……………………………….....SI 21

Figure S9: ^{1}H-^{1}H DQ-SQ NMR spectrum of **NO_2-PDI** collected at 100 kHz spinning speed at room temperature, using the BABA pulse sequence with a short recoupling time of one rotor periods……………………………..………………………SI 22

Figure S10: The ^{1}H-^{1}H DQ-SQ quantum correlation spectra collected at 100 kHz with a recoupling time of 20 μs. The correlation among aromatic protons is challenging to discern due to the broad peak widths.…………………………………………SI 22

Figure S11: The two dimensional $^{1}H\{^{13}C\}$ CP-HETCOR NMR spectra of the **NO_2-PDI** sample obtained at a contact time of 0.128 ms with a spinning frequency of 12 kHz. The spectra at short mixing time indicates all the carbons attached to proton…………………………………………………………………….....SI 23

Figure S12: The $^{1}H\{^{13}C\}$ CP-HETCOR spectra collected at 60 kHz spinning at room temperature with short contact time of 0.25 ms A) aromatic and B) aliphatic region…………………………………………………………………….....SI 23

Figure S13: The one-dimensional CP-MAS (cross polarization magic angle spinning) spectra of **NO_2-PDI** obtained at 0.128 ms and 4 ms. The CP-MAS spectra at long contact time gives indication about the carbon that are far away from the proton. Set of new correlation peaks start observing at long contact time could be either inter or intra molecular correlations. ……………………………………………………....SI 24

Figure S14: Overlay of all 286 predicted conformers of **NO_2-PDI** from the molecular mechanics search…………………………………………………….……………SI 24

Figure S15: The four cases obtained from 3D electron diffraction used in the solid-state NMR studies.……………………………………………………………....SI 25

Figure S16: The schematic representation of X-aggregate stacking of **NO_2-PDI** showing the dihedral angle as 45.72 degrees between the two monomers……..SI 25

Figure S17: Energy comparison of X-aggregate and hypothetical J-aggregate...SI 26

Figure S18: Excitation spectra of **NO_2-PDI** in solid-state (powder-black line) and in monomer concentration (solution-red line). Emission spectra of **NO_2-PDI** in solid-state (powder-black dotted line) and in monomer concentration (solution-red dotted line)……………………………………………………………………..……SI 26

Figure S19: Electrostatic surface potential (ESP) maps (0.001 isodensity surface) of **NO_2-PDI** in Top and Side view computed at cam-b3lyp/6-311+g(d,p) level of theory…………………………………………………………………....SI 27

Figure S20: Comparison of simulated and experimental PXRD pattern and solid-state NMR chemical shifts………………………………………………………………SI 27

Section 4: References………………………………………………………………SI 28

Section 1: Materials and Methods

Materials: The samples were synthesized as in the literature, and characterization was done with solution-state NMR, elemental analysis, and IR spectroscopy.[1]

3D electron diffraction: 3D Electron diffraction (3D ED) experiments were performed on an FEI Tecnai G^2 20 microscope (200 kV, λ = 0.0251 Å) with a LaB_6 cathode equipped with a Cheetah ASI direct detection camera (16 bit). The temperature of measurement was 100 K (sample holder tip temperature). Data were measured by 3D electron diffraction method using continuous rotation with integration semi-angle of 0.15°. The powder was directly deposited on the Cu TEM grid. Data were processed with PETS2.[2] Optical distortions were compensated using calibrated values.[3]

Solution-State NMR: The solution-state NMR chemical shift of the monomer is assigned with the help of ^{1}H, ^{1}H-^{1}H COSY (correlation spectroscopy), ^{1}H-^{13}C HSQC (heteronuclear single quantum correlation), and ^{13}C NMR (Figure S2-4). The ^{1}H-^{1}H COSY is used to get the proton correlation, whereas the ^{1}H-^{13}C HSQC helped to get information on proton-attached carbon. With the above-mentioned information, all the carbon atoms are assigned in the ^{13}C solution-state NMR spectra. To understand the solid-state packing of **NO_2-PDI**, solid-state NMR is carried out at room temperature. The ^{13}C solid-state NMR chemical shift (Figure S4) is assigned with solution-state NMR results. Due to the intricate overlapping of the ^{13}C signals in the aromatic region, the assignment of quaternary carbon atoms is challenging. We must remember that the molecule's supramolecular packing could somewhat influence the chemical shift of quaternary carbons on the core region. The CH_3 and CH_2 groups in the alkyl tail have a solid-state NMR chemical shift of 11 ppm and 24 ppm, respectively, with a small difference of 1 ppm compared to the solution-state NMR. In the alkyl tail, the CH group has a unique chemical shift of 58.2 ppm due to the presence of the imide group. The 15C, 15'C, 15aC, 15'aC have an equivalent chemical shift in solid-state and solution-state NMR similarly for 14C, 14'C 14aC, and 14'aC. Like the other carbon atoms in the alkyl tail, 13C and 13aC carbon atoms have identical chemical shifts. The chemical shift of aromatic CH carbons is assigned with the help of one-dimensional cross-polarization experiments (Figure S2-S4). The 5C, 4C, 10aC, and 9aC have a chemical shift of 135, 132, 123, and 135 ppm, respectively in accordance with the corresponding chemical shift observed in the solution-state NMR. Quaternary carbons 7C, 2C, 7aC, 2aC, 3C, 1aC, 1C, and 3aC have a chemical shift from 125 ppm to 135 ppm. The 11C, 12C, 11aC, and 12aC are quaternary carbons with carbonyl group and have a chemical shift in the range of 161.8 to 162.7 ppm, which is in consonance with 163.3 ppm in the solution-state

NMR. Quaternary carbon 4a, with a nitro group, has a unique chemical shift of 146 ppm in the solid-state NMR and 147.75 ppm in the solution-state NMR. Further experiments with high spinning speeds are needed to resolve the overlapping peaks of quaternary carbon atoms. The broad peaks observed at 11 and 24 ppm corresponding to alkyl tails indicate disorder.

Solid-State NMR: All solid-state NMR spectra were measured with a Bruker Avance HD 600 WB NMR spectrometer ($\nu_0(^1H)$ = 600.1 MHz). Finely powdered samples were filled in 0.7 mm, 1.3 mm, and 4.0 mm and spun at MAS rates of 100 kHz, 60 kHz, or 12 kHz, respectively. ^1H and ^{13}C chemical shifts are quoted relative to neat tetramethylsilane (TMS) using adamantane as a secondary chemical shift standard.

<u>Experiments at 12 kHz</u>: All experiments performed at 12 kHz used the 4.0 mm double resonance probe head. The fine powder of the samples was packed in a 4.0 mm outer diameter (o. d.) ZrO_2 rotor. The ^1H-^{13}C ramp-cross-polarization (rampCP)[4] experiments were performed with a ^{13}C nutation frequency of 60 kHz, and the ^1H nutation frequency optimized at the +1 Hartman-Hahn condition[5] ~72 kHz. The repetition delay was 2-3 s. $^1H\{^{13}C\}$ CP-HETCOR experiments were performed with a contact time of 50 μs and 1.0 ms, and 32-128 t_1 increments consisting of 256-512 scans were collected in each experiment. SPINAL64 decoupling[6] (^1H B_1 field of ~83 kHz) was applied during acquisition. DUMBO[7] homonuclear decoupling schemes were employed during the t_1 evolution at ~100 kHz. The ^1H scaling factor for DUMBO HETCOR was corrected using alanine as the standard, and ^{13}C shift scale were calibrated with adamantane as an external standard.

<u>Experiments at 60 kHz in 1.3 mm probe</u>: Single-pulse ^1H spectra were recorded using 90° rf pulses operating at the ^1H nutation frequency $\nu_H \approx 185$ kHz, 16 accumulated NMR-signal transients, and relaxation delays (τ_{relax}) of 2.0 s. The ^{13}C spectra were recorded by the $^1H \rightarrow {}^{13}C$ CP at the double quantum Hartmann–Hahn condition,[5] $\nu_H + \nu_C = \nu_r$, which involved ramped CP of $\nu_H = 20 \pm 5$ kHz for ^1H ($\nu_C = 40$ kHz), a 1.5 μs 90° ^1H pulse, and spinal-64 ^1H decoupling[8] at νH = 150 kHz. The $^{13}C\{^1H\}$ 2D HETCOR NMR spectra were recorded with $\tau_{CP} = 250$ μs, and 1.0 ms $\tau_{relax} = 2.0$ s, and dwell times of $\Delta t_2 = 22.0$ μs and $\Delta t_1 = 6\tau r = 100$ us, where $\tau_r = \nu r^{-1}$ is the rotor period. $32(t_1) \times 2048(t_2)$ time points were collected with 2048 (τ_{CP} =1.0 ms) and 8192 (τ_{CP} = 100 μs) accumulated transients per t_1 value. The 2D NMR data sets were zero-filled to 64 t_1 points, along with 4096 (t_2), and were apodized by a $\cos^2$ and an exponential function along the indirect and direct dimensions, respectively, with the latter giving a 50 Hz full width at half-maximum (fwhm) Lorentzian broadening.

The DQ−SQ ^{1}H NMR correlation spectra were recorded with the 2D NMR protocol shown in Figure 1a of ref [9]. The 2Q coherence (2QC) excitation/reconversion was accomplished by the BaBa dipolar recoupling scheme that extends over one sole rotor period,[9] thereby giving the shortest 2Q excitation (τ_{exc}) and reconversion (τ_{rec}) intervals of $\tau_{exc} = \tau_{rec} = \tau_r = 16.66$ μs. The ^{1}H nutation frequency was $\nu_H \approx 185$ kHz for the 90° dipolar recoupling pulses of a duration of 1.35 μs. The 2D NMR acquisitions employed $\tau_{relax} = 2.0$ s, 64(t_1) × 512(t_2) time points were acquired with dwell times of {$\Delta t_1 = 3\tau_r$; $\Delta t_2 = 2.0$ μs} and 64 accumulated transients/t_1-value. The 2D data sets were zero-filled to 256 × 2048 points and apodized by an exponential 50 Hz fwhm Lorentzian broadening was applied both dimensions.

Experiments at 100 kHz: - For all experiments in the 0.7 mm MAS probe the ^{1}H π/2 and π pulse lengths were 0.7 and 1.4 μs in duration, corresponding to a 360 kHz RF field.

T1: The ^{1}H spin lattice relaxation times were measured using a saturation recovery experiment. Four scans were collected with a relaxation delay of 5s with τ delays varying from 0.5 ms to 100 s in a pseudo 2D mode. The data was analyzed with the help of Bruker T_1/T_2 analytical routine.

BABA: The DQ−SQ ^{1}H NMR correlation spectra also recorded at 100 kHz MAS were the 2Q excitation (τ_{exc}) and reconversion (τ_{rec}) time was $n\tau_r$ where n=1, or 2. The ^{1}H nutation frequency was $\nu_H \approx 360$ kHz for the 90° dipolar recoupling pulses of a duration of 0.7 μs. The 2D NMR acquisitions employed $\tau_{relax} = 5.0$ s, 44(t_1) × 512(t_2) time points were acquired with dwell times of {$\Delta t_1 = 4\tau_r = 40.0$ μs; $\Delta t_2 = 20.0$ μs} and 64 accumulated transients/t_1-value. The 2D data sets were zero-filled to 256 × 2048 points and apodized by an exponential 50 Hz fwhm Lorentzian broadening was applied both dimensions.

DFT calculation: The FORCITE module in the Materials studio was used for the initial relaxation of the structures with the Universal force field. Geometry optimizations were performed using the Smart algorithm with a convergence tolerance energy of 0.0001 kcal mol^{-1} and force of 0.005 kcal Å^{-1} with a maximum number of iterations of 500. Plane-wave DFT calculations were done with the gauge-including projected augmented wave (GIPAW)[10,11] approach as implemented in the CASTEP[12] version 2017. Geometry optimization and NMR properties were calculated using the generalized gradient approximation (GGA) with the exchange-correlation PBESOL functional,[13] with On-the-Fly ultra-Pseudopotential.[14] And the Tkatchenko and Scheffler method was employed for dispersion corrections.[15] An energy cutoff of 630 eV with a Monkhorst−Pack grid[16] with a k-point spacing of 0.07 Å^{-1} was chosen to maximize the calculation efficiency.

Molecular electrostatic surface potential (ESP) maps were calculated from the cube files generated by Gaussian 13 energy calculations of **NO_2-PDI** using GaussView 5.0.8 software.[17]

Symmetry Adapted Perturbation Theory (SAPT) Analysis: Symmetry Adapted Perturbation Theory (SAPT): SAPT(0) analysis was employed to determine the non-covalent interaction energies of dimer molecules. The SAPT module of the psi4 code was employed, with aug-cc-pVDZ basis set. SAPT(0) calculations provide the contributing components of interaction energy. The results obtained from SAPT(0) analysis is a second order perturbation expansion constituting first order electrostatic ($E^{(1)}_{elc}$) and exchange energy ($E^{(1)}_{ex}$) parts, and second order dispersion ($E^{(2)}_{dis}$), induction ($E^{(2)}_{ind}$) and their exchange counterparts as the perturbation terms,

$$E^{SAPT(0)}_{int} = E^{(1)}_{elc} + E^{(1)}_{ex} + E^{(2)}_{ind} + E^{(2)}_{ind-ex} + E^{(2)}_{dis} + E^{(2)}_{dis-ex}$$

Photoluminescence Measurements: Photophysical measurements of the derivatives were carried out in a quartz cover slip. The powder sample was packed inside a quartz cover slip and used for the measurements. Emission and excitation spectra were recorded on Horiba Jobin Yvon Fluorolog spectrometer.

PXRD: Powder X-ray diffraction pattern was collected using Rigaku Smartlab X-ray diffractometer with Cu Kα radiation.

Density Measurement: The experiments were performed with a Micrometrics Accupyc II 1340 Gas Displacement Pycnometer System with temperature control and a 100 cm^3 nominal cell volume containing 10 cm^3 cups. For each analysis 0.152 g were weighed directly into the cup. The cup with the sample was placed inside the cell, which thereafter was placed inside the sample compartment and sealed. Nitrogen was passed into the sample compartment until reaching an equilibrium rate of 0.0050 psig/min. After pressure stabilization, gas was allowed to expand into the reference compartment. The pressure before and after expansion was measured automatically and used to compute the sample volume. The ratio of known sample mass and volume yields the density. Each analysis took between 45 mins to 1 hour with pressure equilibration being the most time-consuming step.

Conformer Distribution Search: Given the significant conformational flexibility of **NO_2-PDI**, the set of low energy conformers of this molecule were determined by performing a conformer distribution search using Spartan'18. The conformer distribution search finds the equilibrium

geometry of each conformer by considering rotations around single bonds and puckering of the rings. The calculation was performed using the MMFF molecular mechanics model. During the search a maximum of 500 conformers were requested and only conformers within a relative energy of 40 kJ mol^{-1} were accepted. The search led to a total of 286 conformers, which were overlayed (Figure S20) to provide a qualitative assessment of the origins of conformational disorder in this molecule.

Section 2: Tables

Table S1: Packing of related molecule from the literature.

Sl no	Name	Space group	Lattice type	Aggregate	
1	PTE[18]	4 *C*2/c	Monoclinic	X-aggregate	
2	NO_2-PDI	61 *Pbca*	Orthorhombic	X aggregate	This work
3	Bis nitro PDI[19,20]	9 *Cc*	Monoclinic	J aggregate	

Table S2: Summary of the information obtained from various techniques.

Technique	Information obtained for current work
3D ED	Type of aggregate, conformation of side chain groups, packing
solid-state NMR	Spatial correlation between tails and perylene core, distance between perylene core and alkyl tail, disorder exist in the tail
Photoluminescence measurement	X-aggregate
PXRD	Pattern matching using simulated powder pattern from model provided by 3D ED
SAPT, DFT calculation	X-aggregate, alignment of dipole moment
CASTEP simulation	Confirmation of the structure

Table S3: Density of the related compounds from the literature.

Sl no	Name of the compound	Unit cell parameters						σ
		a(Å)	b(Å)	c(Å)	α (°)	β(°)	γ(°)	(gm/cm^3)
1	N,N′-bis(3-pentyl)- perylene-3,4,9,10-bis(dicarboximide)	33.90	7.33	21.10	90.00	100.33	90.00	1.37[18]
2	Naphthalene-1,8:4,5-bis(dicarboximide)	9.76	17.27	20.76	67.36	79.09	82.90	1.07[21]
3	Perylene Diimide-Embedded Double [8]Helicenes	15.14	19.49	22.41	106.70	98.60	105.02	1.15[22]
4	Perylene-bisimide, N,N′-di-(n-butyl)-1,7-dicyclopentadienyl-cobalt(II)-dithiolenyl-perylene-3,4:9,10-tetracarboxylic acid bisimide	30.96	15.40	21.15	90.00	117.24	90.00	1.29[23]
5	Monobay-dichlorinated perylene bisimide	8.58	33.29	15.73	90.00	103.60	90.00	1.24[24]
6	1,7-dibromoperylene bisimide	10.59	11.26	14.36	80.29	68.79	89.14	1.55[25]
7	Alkylated N-annulated PDI	13.79	37.42	7.30	90.00	98.43	90.00	1.28[26]
8	N,N′-bis(carboxyethyl)perylene-3,4,9,10-tetracarboxylic bisimide	10.86	11.86	17.71	92.06	93.78	114.52	1.45[27]

Table S4: Experimental and calculated ^{13}C -NMR chemical shift of the **NO_2-PDI** molecule.

Position	δ_{solid} expt, ppm	$\sigma_{solid*\ calc,}$	$\delta_{\sigma olid}$-$\sigma_{solid*\ calc}$, ppm
C11a	163.6	167.2	-3.6
C11	162.9	163.5	-0.6
C12a	162.3	166.3	-4.0
C12	161.5	165.2	-3.7
C4a	146	139.9	6.1
C9a	134.3	130.6	3.7
C10	132.9	125.9	7.0
C9	132.2	138.2	-6.0
C1a	132.1	134.2	-2.1
C7	130.7	122.9	7.8
C10a	129.5	128.3	1.2
C3	129.1	131.9	-2.8
C7a	128.6	127.5	1.1
C5	127.7	120.2	7.5
C5a	127.1	129.7	-2.6
C8a	126.5	119.3	7.2
C1	126.1	125.9	0.2
C2	125.1	124.5	0.6
C8	124.7	119.6	5.1
C4	123.5	123.4	0.1
C6	123.4	123.3	0.1
C2a	123.4	128.4	-5.0
C3a	121.6	117.8	3.8
C6a	121.2	125.5	-4.3
C13a	59.3	57.4	1.9
C13	59.06	61.7	-2.7
C14a	28.3	24.9	3.4
C14'	26.1	26.3	-0.2
C14	25.1	27.6	-2.5
C14'a	24.1	25.9	-1.8
C15	12.7	16.0	-3.3
C15a	12.3	17.43	-5.1
C15’	11.4	15.15	-3.7
C15	10.7	13.25	-2.5
			RMSD = 4.00

Table S5: The ^{1}H-NMR chemical shifts of **NO_2-PDI**.

Position		δ_{soln}, ppm	δ_{solid}, ppm	σ_{calc}, ppm
5	CH	8.64	8.62	9.18
9a	CH	8.67	8.83	8.52
9	CH	8.52	8.44	8.18
10	CH	8.18	7.14	7.10
10a	CH	8.72	7.20	6.35
5a	CH	8.64	6.97	6.01
13	CH	4.98	5.19	5.72
4	CH	8.65	6.45	5.40
13a	CH	4.98	4.32	4.60
14'a_2	CH_2	1.87	2.84	2.92
14'a_1	CH_2	2.17	1.87	2.28
14_1	CH_2	2.17	2.50	2.50
14_2	CH_2	1.87	1.70	1.79
14a_1	CH_2	2.17	2.37	2.33
14a_2	CH_2	1.87	1.82	2.15
14'_1	CH_2	2.17	1.60	1.57
14'_2	CH_2	1.87	1.87	2.80
15'a	CH_3	0.85	1.50	1.43
15'	CH_3	0.85	0.90	1.38
15a	CH_3	0.85	1.11	1.20
15	CH_3	0.85	0.91	0.82

Table S6: The experimental chemical shift of ^{1}H and calculated chemical shift of four different cases

numbering scheme	$\delta_{expt,ppm}$	$\delta_{calc,ppm}$	δ_{expt}-δ_{calc}	$\delta_{calc,flipped\ short\ axis,ppm}$	δ_{expt}-$\delta_{calc,flipped\ short\ axis,ppm}$	$\delta_{calc,methyl\ rotated,ppm}$	δ_{expt}-$\delta_{calc,methyl\ rotated,ppm}$	$\delta_{calc,flipped\ long\ axis,ppm}$	δ_{expt}-$\delta_{calc,flipped\ long\ axis,ppm}$
5H	8.62	9.18	-0.56	7.73	0.89	6.16	2.46	8.63	-0.01
9aH	8.83	8.52	0.31	5.66	3.17	8.48	0.35	6.81	2.02
9H	8.44	8.18	0.26	7.2	1.24	8.55	-0.11	5.23	3.21
10H	7.14	7.1	0.04	6.04	1.1	5.44	1.7	5.87	1.27
10aH	7.2	6.35	0.85	5.7	1.5	7.81	-0.61	7.82	-0.62
5aH	6.97	6.01	0.96	8.39	-1.42	6.58	0.39	8.08	-1.11
13H	5.19	5.72	-0.53	4.51	0.68	7.07	-1.88	4.59	0.6
4H	6.45	5.4	1.05	6.81	-0.36	5.65	0.8	9.06	-2.61
13aH	4.32	4.6	-0.28	5.62	-1.3	5.13	-0.81	4.98	-0.66
14'a_2H	2.84	2.92	-0.08	2.43	0.41	2.66	0.18	2.01	0.83
14'a_1H	1.87	2.28	-0.41	1.65	0.22	1.91	-0.04	2.28	-0.41
14_1H	2.5	2.5	0	2.2	0.3	2.53	-0.03	2.3	0.2
14_2H	1.7	1.79	-0.09	2.68	-0.98	1.84	-0.14	2.42	-0.72
14a_1H	2.37	2.33	0.04	2.6	-0.23	2.62	-0.25	2.47	-0.1
14a_2H	1.82	2.15	-0.33	1.65	0.17	2.63	-0.81	2.53	-0.71
14'_1H	1.6	1.57	0.03	1.54	0.06	1.22	0.38	2.47	-0.87
14'_2H	1.87	2.8	-0.93	2.34	-0.47	2.89	-1.02	3	-1.13
15'aH	1.5	1.43	0.07	1.39	0.11	1.05	0.45	1.01	0.49
15'H	0.9	1.38	-0.48	1.45	-0.55	1.53	-0.63	1.03	-0.13
15aH	1.11	1.2	-0.09	0.81	0.3	0.86	0.25	1.68	-0.57
15H	0.91	0.82	0.09	1.94	-1.03	1.42	-0.51	1.5	-0.59

Table S7: Solution and solid-state ^{13}C-NMR chemical shift assignments of the **NO_2-PDI** molecule.

Position	δ_{soln}, ppm	δ_{solid}, ppm	δ_{soln} - δ_{solid}, ppm
C11a	164.02	163.6	0.42
C11	164.10	162.9	1.2
C12a	161.94	162.3	-0.36
C12	162.76	161.5	1.26
C4a	147.75	146	1.75
C9a	133.01	134.3	-1.29
C10	131.31	132.9	-1.59
C9	127.91	132.2	-4.29
C1a	127.53	132.1	-4.57
C7	129.13	130.7	-1.57
C10a	124.49	129.5	-5.01
C3	132.93	129.1	3.83
C7a	123.07	128.6	-5.53
C5	124.12	127.7	-3.58
C5a	126.43	127.1	-0.67
C8a	129.53	126.5	3.03
C1	124.93	126.1	-1.17
C2	126.48	125.1	1.38
C8	124.54	124.7	-0.16
C4	126.62	123.5	3.12
C6	124.18	123.4	0.78
C2a	129.35	123.4	5.95
C3a	135.50	121.6	13.9
C6a	132.72	121.2	11.52
C13a	58.23	59.3	-1.07
C13	57.94	59.06	-1.12
C14a	24.97	28.3	-3.33
C14'	24.91	26.1	-1.19
C14	24.91	25.1	-0.19
C14'a	24.97	24.1	0.87
C15	11.30	12.7	-1.4
C15'a	11.32	12.31	-0.99
C15a	11.32	11.42	-0.1
C15'	11.30	10.71	0.59

Table S8: Dipole moment of the monomer and X-dimer of **NO_2-PDI** computed at cam-b3lyp/6-311+g(d,p) level of theory.

Monomer/X-Dimer	Dipole Moment (Debye)
Monomer	5.26
X-Dimer	3.59

Table S9: Total intermolecular interaction (SAPT(0)) energies and its components for X-dimer.

Electrostatics (kJ/mol)	Exchange (kJ/mol)	Induction (kJ/mol)	Dispersion (kJ/mol)	Total Interaction Energy (kJ/mol)
-37.55	106.92	-18.23	-211.94	-160.81

Table S10: Excitation and emission properties of **NO_2-PDI** in solution-state (monomer) and solid-state (aggregate).

**NO_2-PDI**	Excitation (λ_{max}^{Exc}, nm)	Emission (λ_{max}^{Emi}, nm)
Solution-state	520	536
Solid-state	586	653

Section 3: Figures

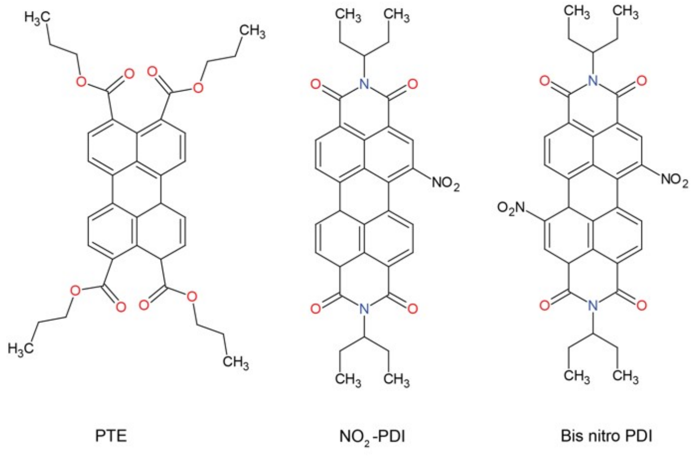

Figure S1: The structure of **NO_2-PDI** and related molecules.

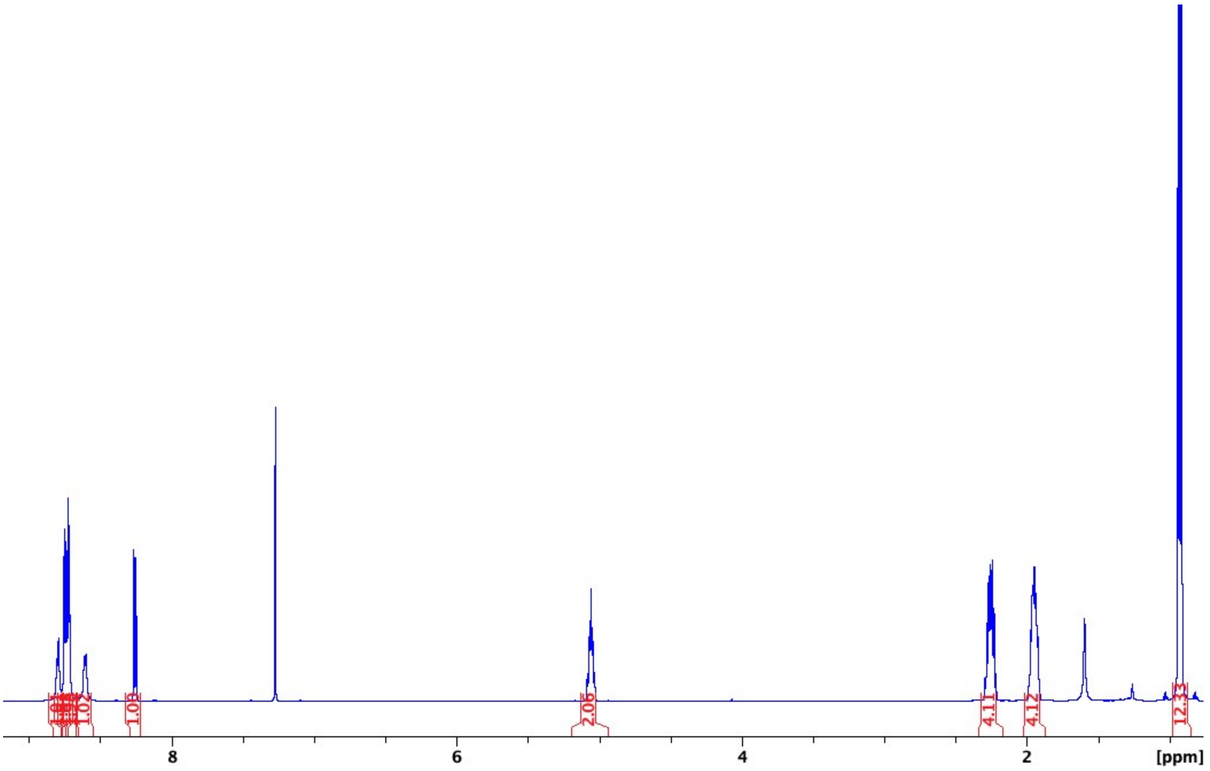

Figure S2: ^{1}H-NMR spectra of the sample collected at room temperature on 500 MHz instrument with $CDCl_3$ solvent.

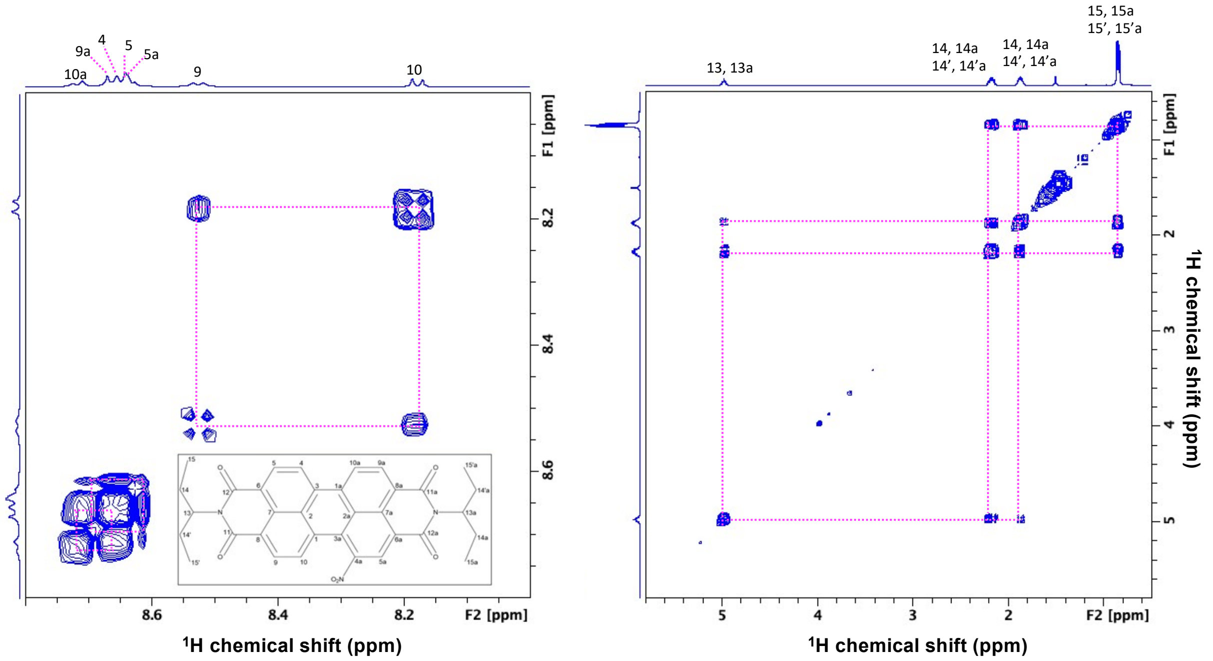

Figure S3: ^{1}H-^{1}H COSY spectrum of the sample collected at room temperature on 500 MHz instrument with $CDCl_3$ solvent..

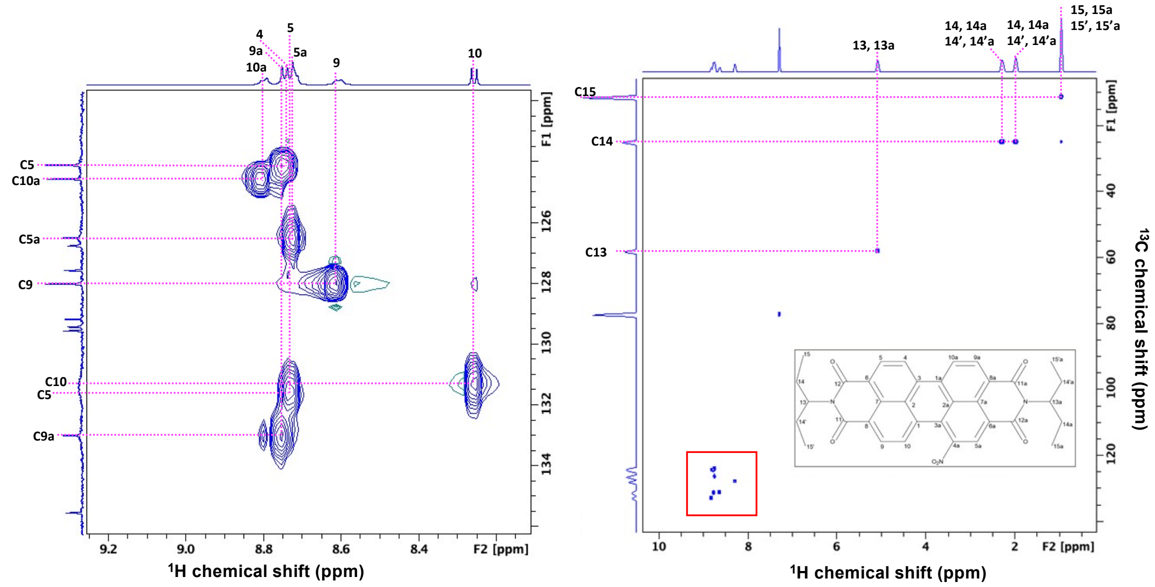

Figure S4: The ^{1}H-^{13}C HSQC spectra of the sample showing all the CH carbon atoms collected at room temperature with $CDCl_3$ solvent.

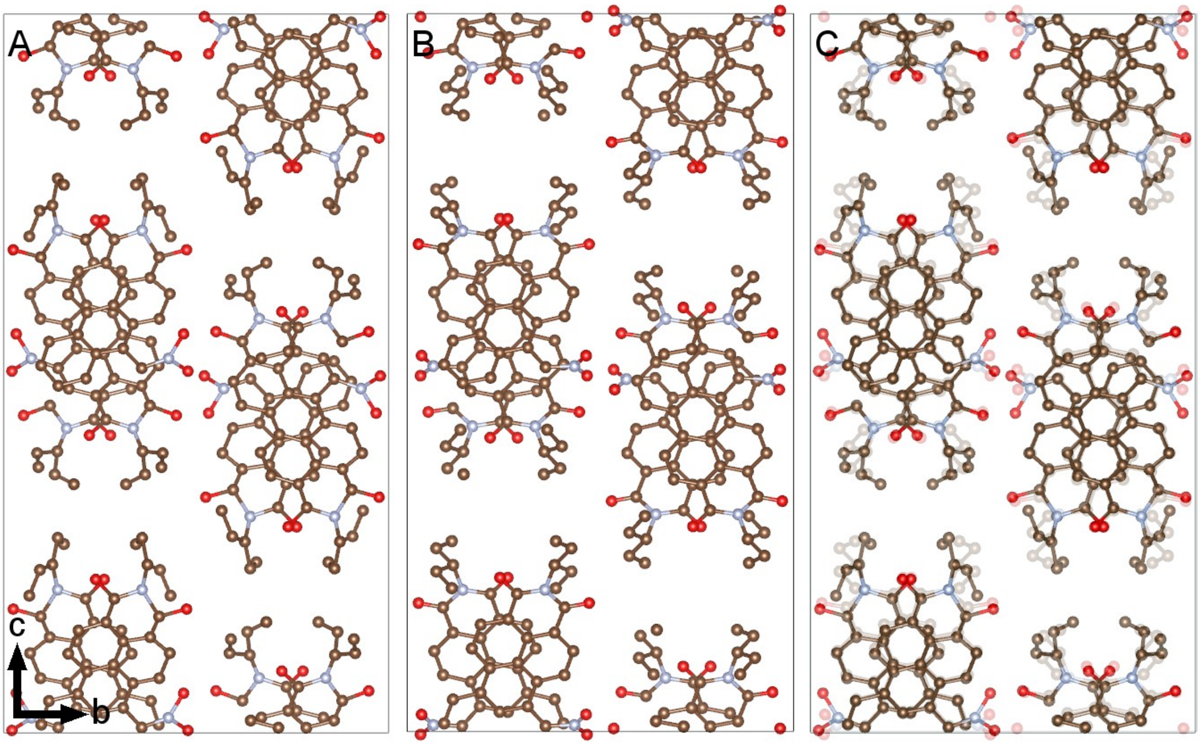

Figure S5: Solution of **NO_2-PDI** (A), optimized molecule in Molview inserted into the place of the solved molecule position (B), overlay of the two structures (C, optimized molecule with one third of the intensity of the solution).

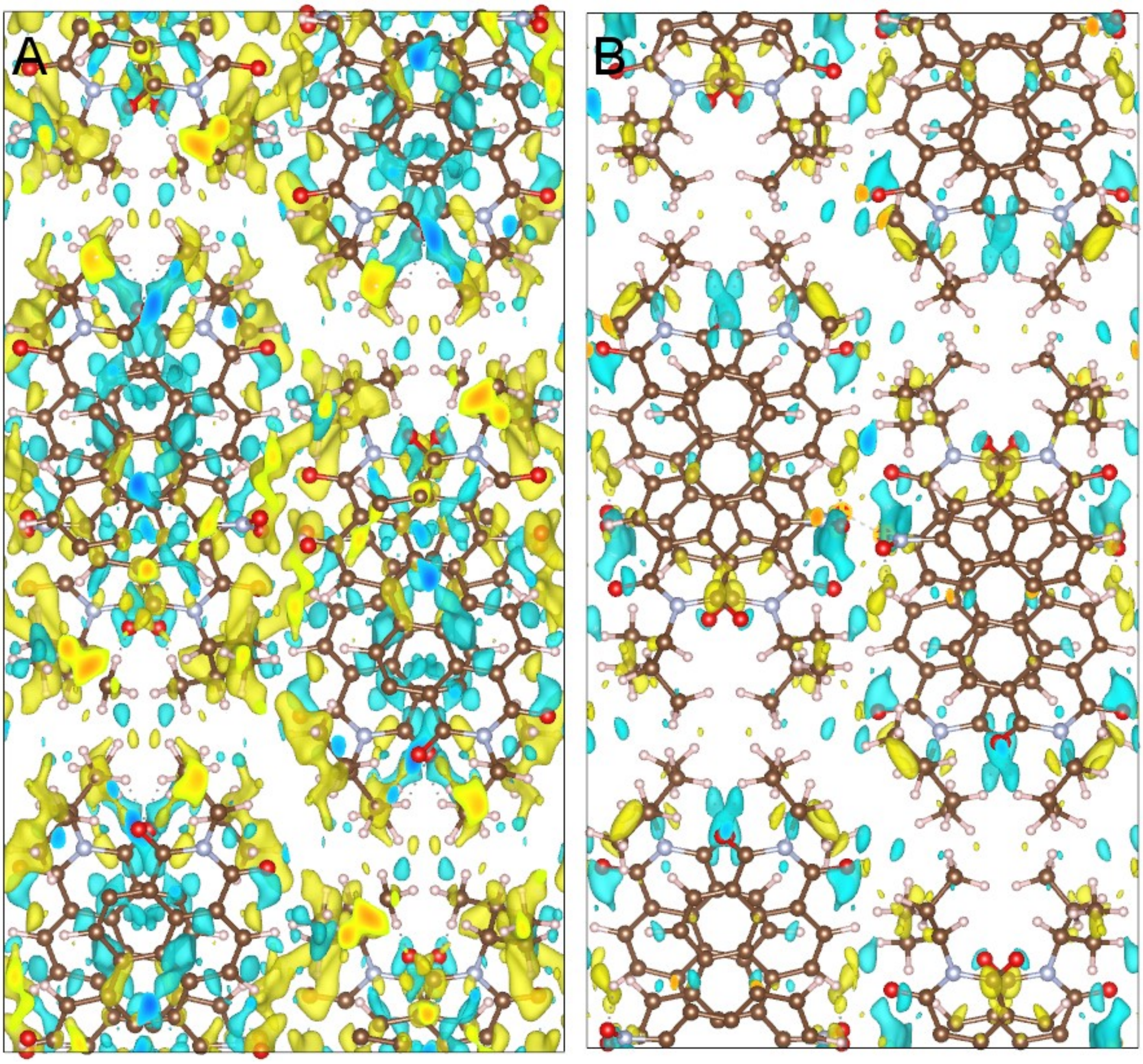

Figure S6: Difference potential at 2 sigma level after constrained core refinement with anisotropic ADPs with inserted MolView optimized molecule to best represent the difference potential features in the peripheral parts of the molecule (A). Difference potential at 2.5 sigma level after the whole molecule constrained refinement (B). The distance between the oxygen atoms of the neighboring nitro groups (dashed line) is only about 2.0 Å. The absolute value of the isosurface level for both parts of the figure is 0.40 e.Å^{-1}. The unit cells are viewed along [100] direction.

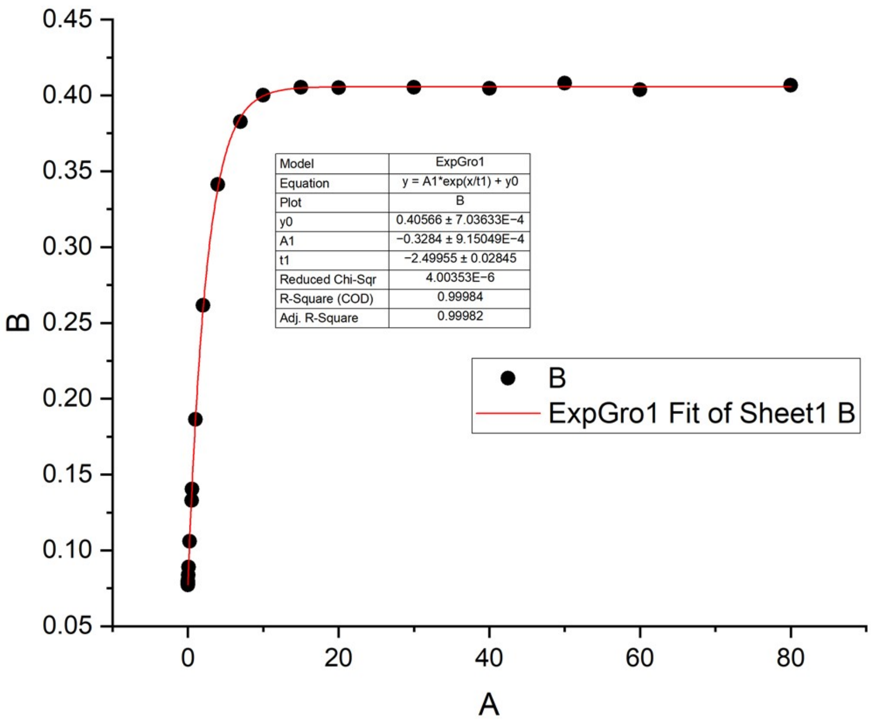

Figure S7: The T_1 measurement with monoexponential fit is shown here. The T_1 of aliphatic tails are short indicating the mobility or disorder of the species at room temperature.

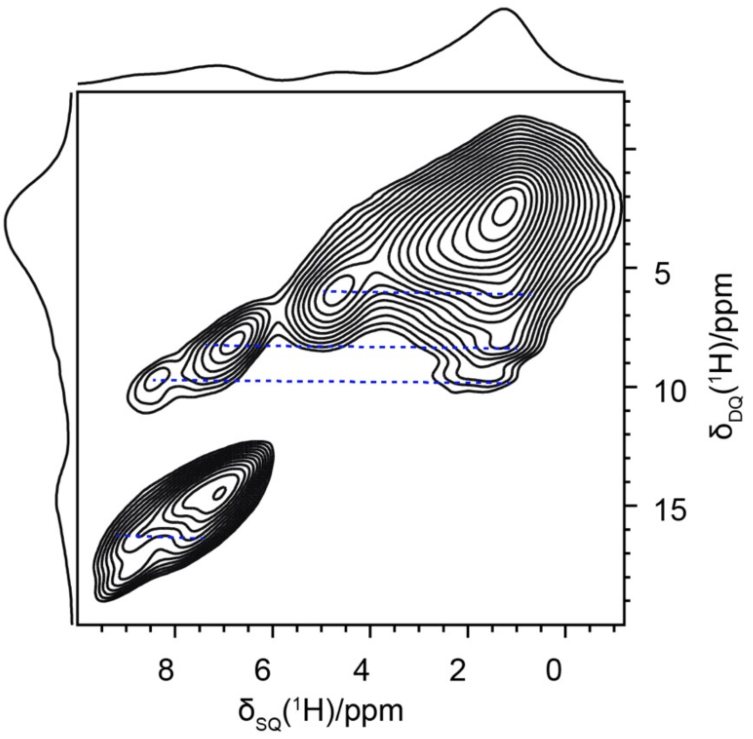

Figure S8: ^{1}H-^{1}H DQ-SQ NMR spectrum of **NO_2-PDI** collected at 60 kHz spinning speed at room temperature, using the BABA pulse sequence with a recoupling time of one rotor periods corresponding to 16 μs.

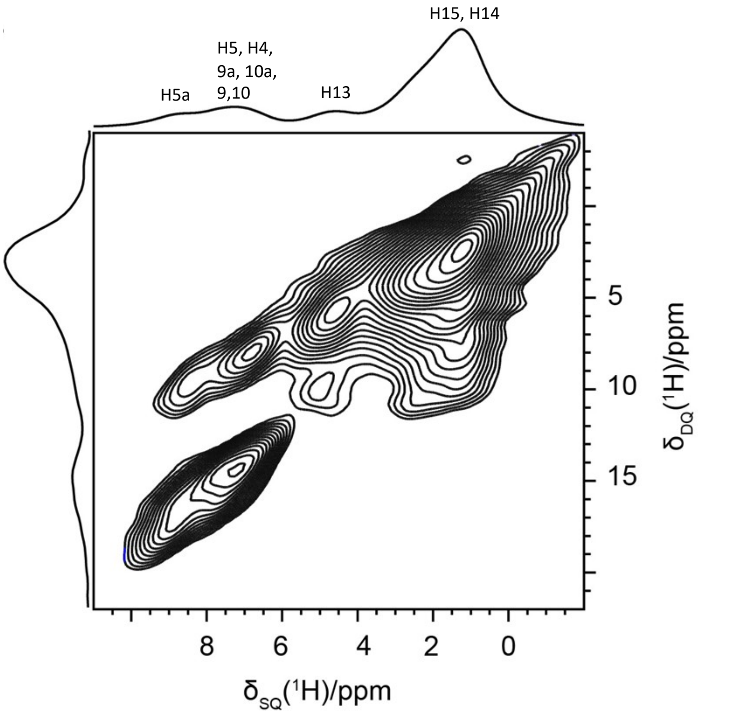

Figure S9: ^{1}H-^{1}H DQ-SQ NMR spectrum of **NO_2-PDI** collected at 100 kHz spinning speed at room temperature, using the BABA pulse sequence with a short recoupling time of one rotor periods.

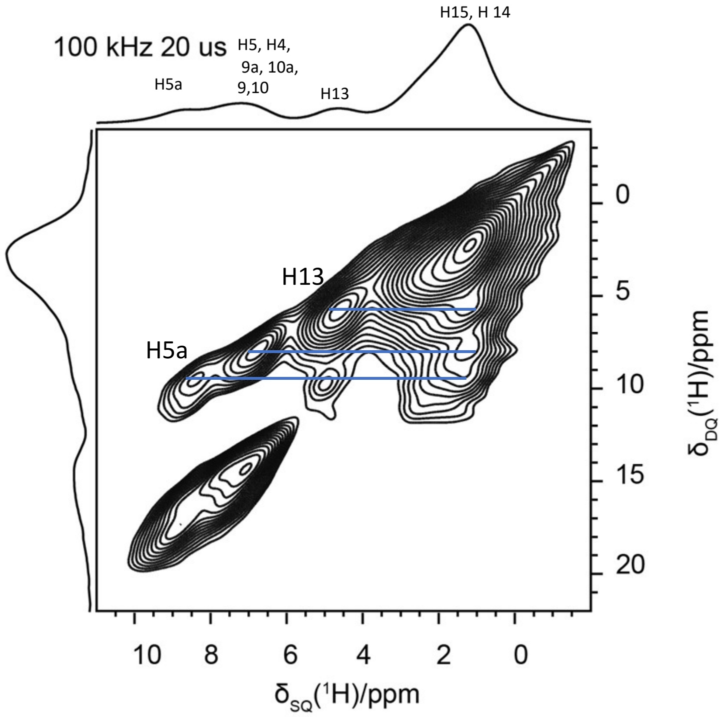

Figure S10: The ^{1}H-^{1}H DQ-SQ quantum correlation spectra collected at 100 kHz with a recoupling time of 20 μs (two rotor periods). The correlation among aromatic protons is challenging to discern due to the broad peak widths.

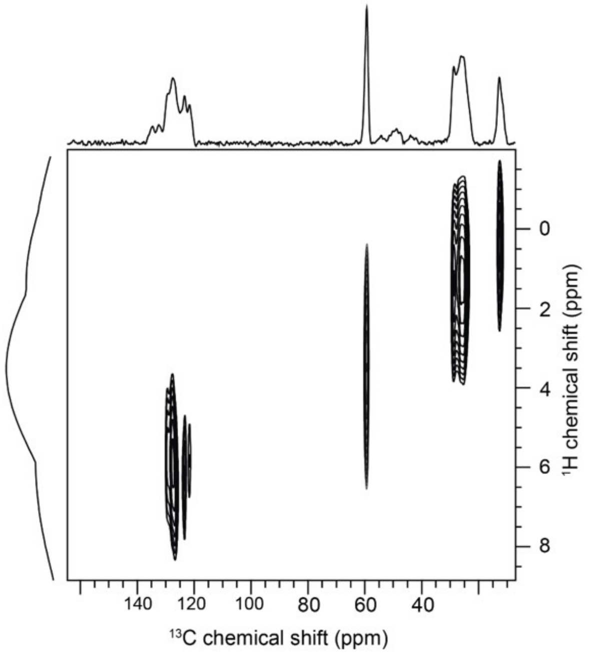

Figure S11: The two dimensional $^{1}H\{^{13}C\}$ CP-HETCOR NMR spectra of the **NO_2-PDI** sample obtained at a contact time of 0.128 ms with a spinning frequency of 12 kHz. The spectra at short mixing time indicates all the carbons attached to proton.

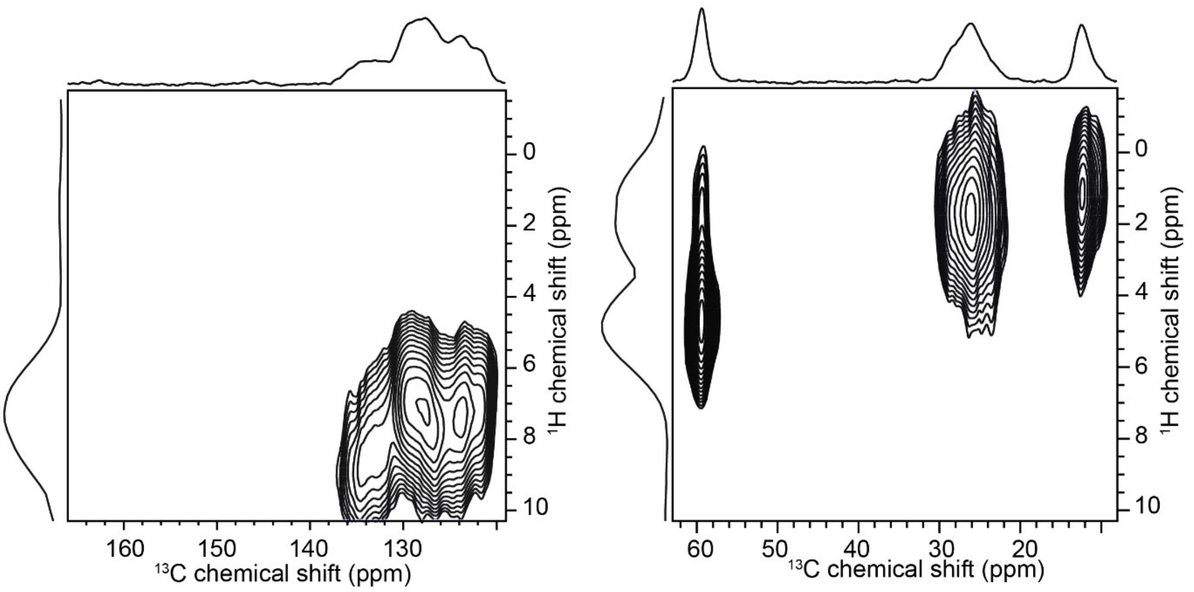

Figure S12: The $^{1}H\{^{13}C\}$ CP-HETCOR spectra collected at 60 kHz spinning at room temperature with short contact time of 0.25 ms A) aromatic and B) aliphatic region.

.

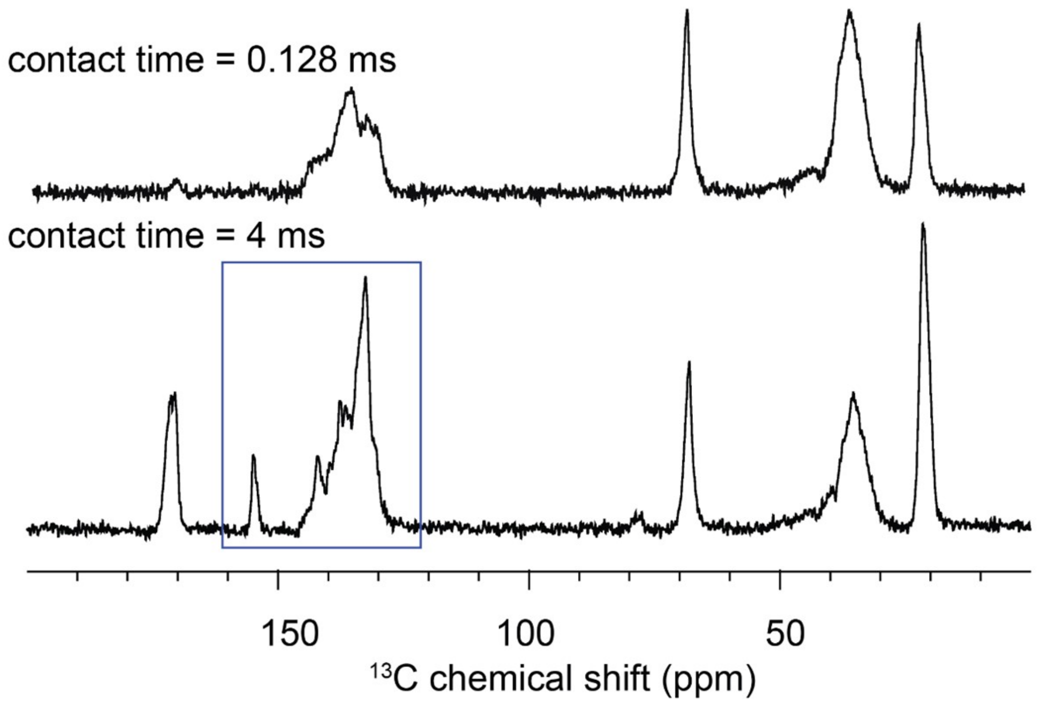

Figure S13: The one-dimensional CP-MAS (cross polarization magic angle spinning) spectra of **NO_2-PDI** obtained at 0.128 ms and 4 ms. The CP-MAS spectra at long contact time gives indication about the carbon that are far away from the proton. Set of new correlation peaks start observing at long contact time could be either inter or intra molecular correlations.

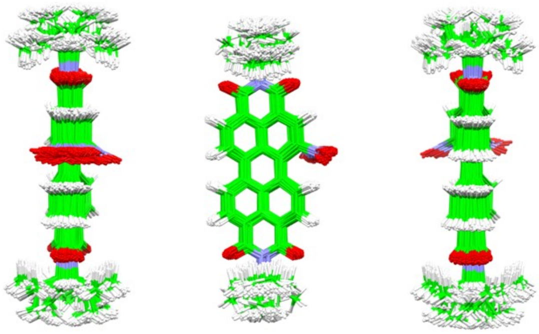

Figure S14: Overlay of all 286 predicted conformers of **NO_2-PDI** from the molecular mechanics search indicating that any conformational disorder in this molecule will likely arise from different orientations of the alkyl tail given the significant conformational variability in the orientation of the alky tails.

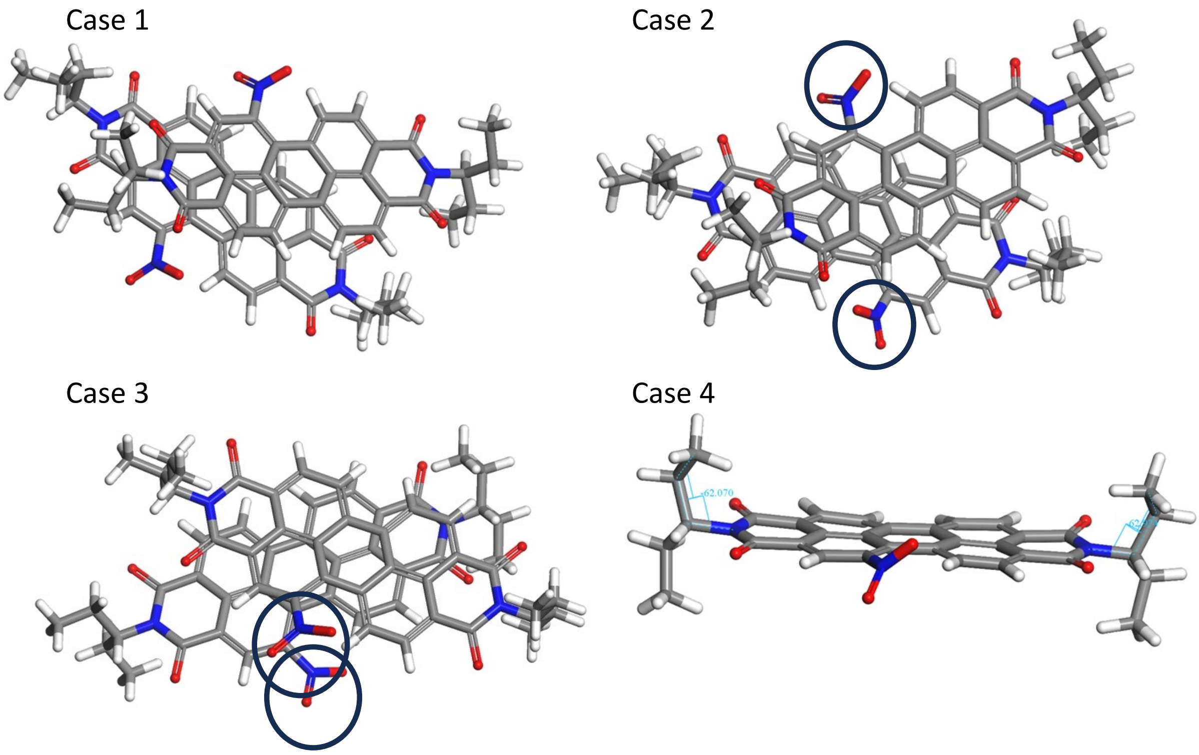

Figure S15: Four cases used in the solid-state NMR studies. Case 1 represents the structure as obtained from 3D electron diffraction and cases 2 to 4 simulate disorder. The molecular conformation is the same in all four cases.

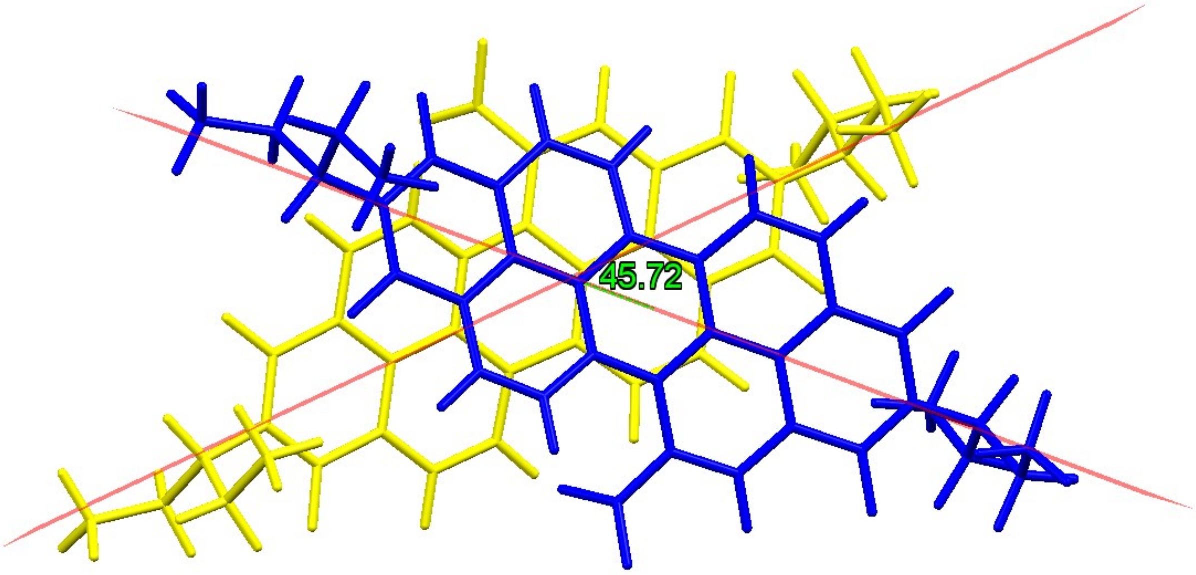

Figure S16: The schematic representation of X-aggregate stacking of **NO_2-PDI** showing the angle between the prependicualr plane of the molecules as 45.72 degrees.

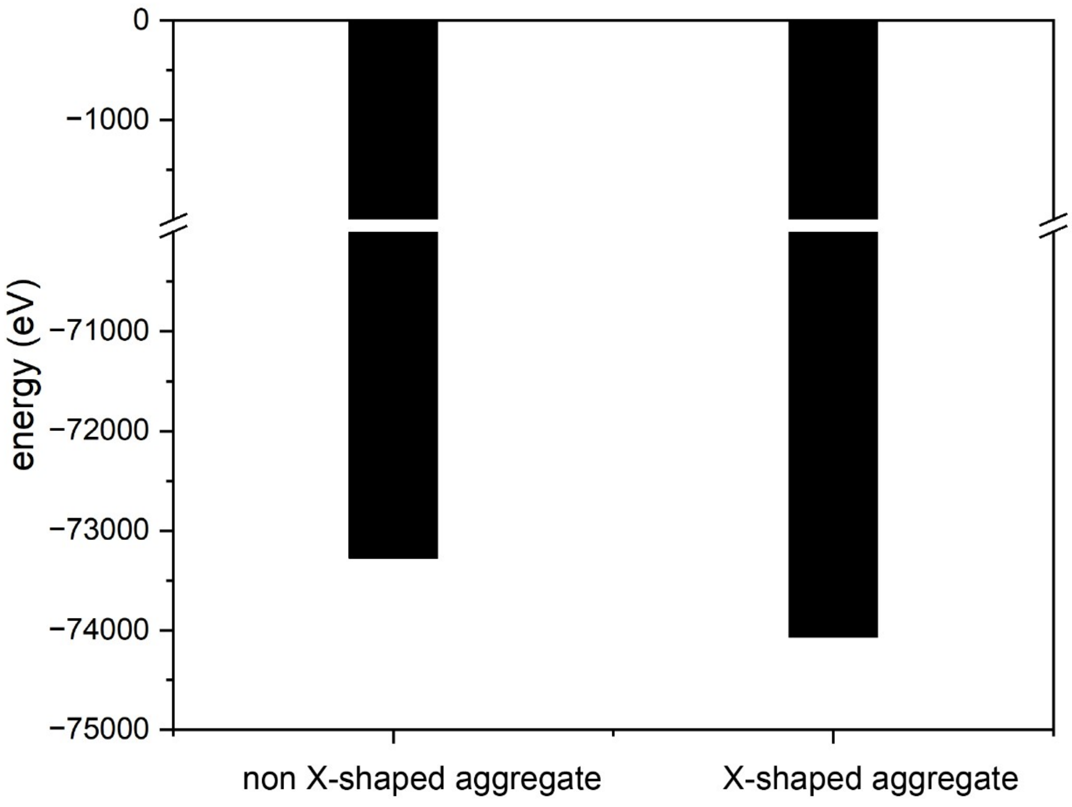

Figure S17: Energy comparison of non X-shaped packing and X- shaped packing.

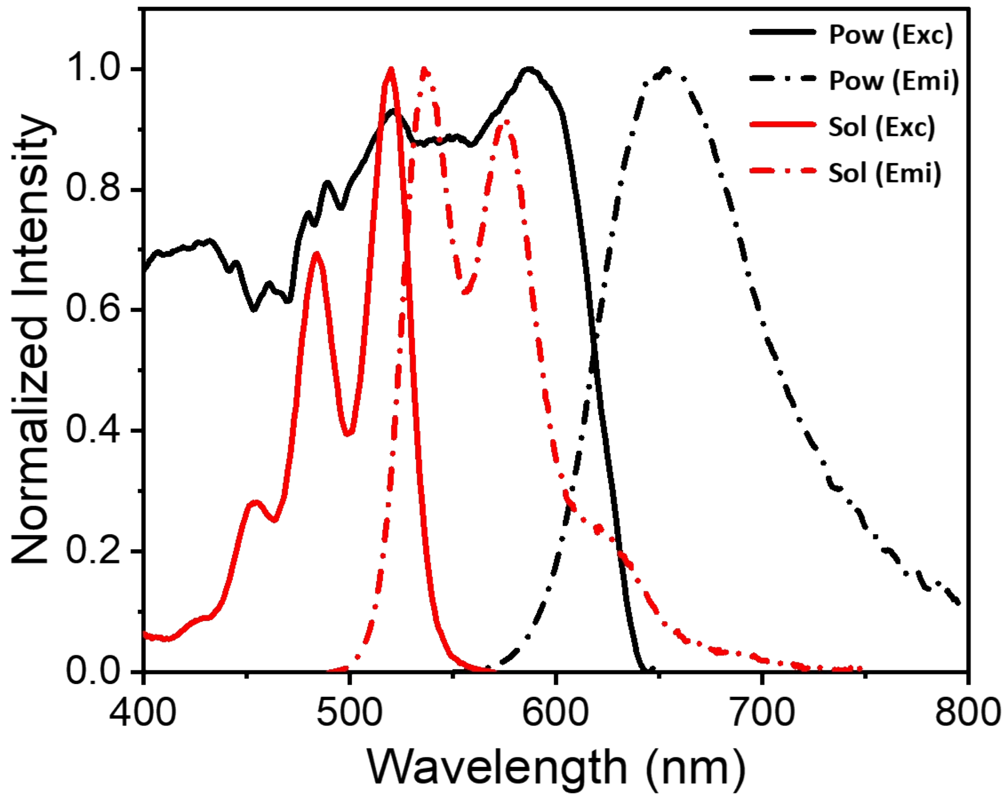

Figure S18: Excitation spectra of **NO_2-PDI** in solid-state (powder- black line) and in monomer concentration (solution- red line). Emission spectra of NO2-PDI in solid-state (powder- black dotted line) and in monomer concentration (solution- red dotted line).

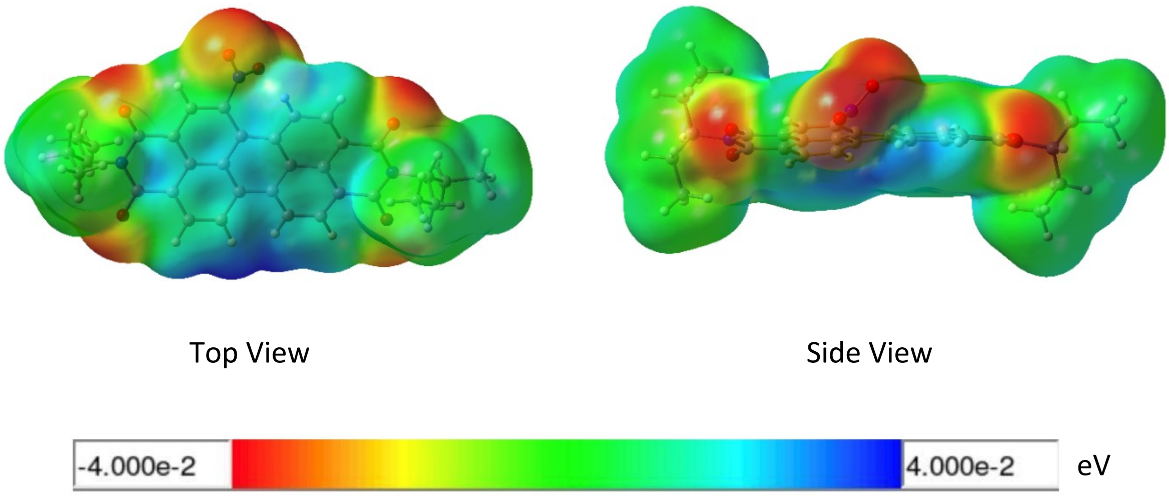

Figure S19: Electrostatic surface potential (ESP) maps (0.001 isodensity surface) of **NO_2-PDI** in Top and Side view computed at cam-b3lyp/6-311+g(d,p) level of theory.

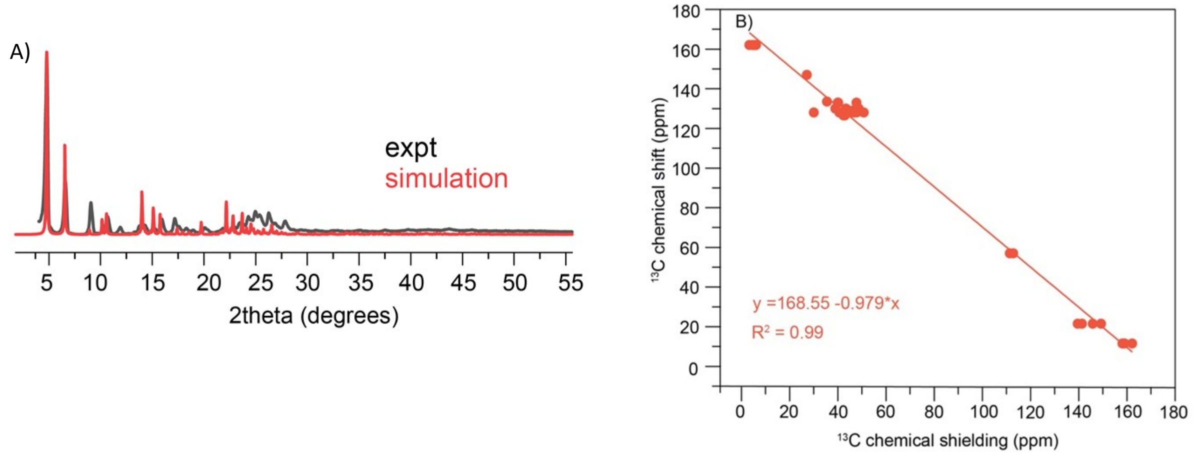

Fig S20: A) The experimental powder XRD pattern (black trace) obtained for the NO_2-PDI molecule at room temperature, along with the predicted powder XRD pattern (red trace) from the derived structure. The powder XRD pattern shows distinct peaks in the low angle region whereas overlapping peaks in the high angle. The sharp peak indicates the crystalline character of the system. B) Correlation of calculated vs experimental ^{13}C isotropic chemical shift of the NO_2-PDI molecule is shown. The calculated chemical shift is obtained from the derived model using CASTEP. A close match is observed between simulated and experimental NMR parameters.

References:

1 A. Mazumder, E. Sebastian and M. Hariharan, *Chem Sci*, 2022, **13**, 8860–8870.

2 L. Palatinus, P. Brázda, M. Jel\'\inek, J. Hrdá, G. Steciuk and M. Klementová, *Acta Crystallographica Section B*, 2019, **75**, 512–522.

3 P. Brázda, M. Klementová, Y. Krysiak and L. Palatinus, *IUCrJ*, 2022, **9**, 735–755.

4 G. Metz, X. Wu and S. O. Smith, *J Magn Reson A*, 1994, **110**, 219–227.

5 W. Kolodziejski and J. Klinowski, *Chem Rev*, 2002, **102**, 613–628.

6 B. M. Fung, A. K. Khitrin and K. Ermolaev, *Journal of Magnetic Resonance*, 2000, **142**, 97–101.

7 D. Sakellariou, A. Lesage, P. Hodgkinson and L. Emsley, *Chem Phys Lett*, 2000, **319**, 253–260.

8 T. G. Oas, R. G. Griffin and M. H. Levitt, *J Chem Phys*, 1988, **89**, 692–695.

9 M. Feike, D. E. Demco, R. Graf, J. Gottwald, S. Hafner and H. W. Spiess, *J Magn Reson A*, 1996, **122**, 214–221.

10 F. Mauri, B. G. Pfrommer and S. G. Louie, *Phys Rev Lett*, 1996, **77**, 5300–5303.

11 C. J. Pickard and F. Mauri, *Phys Rev B Condens Matter Mater Phys*, 2001, **63**, 2451011–2451013.

12 S. J. Clark, M. D. Segall, C. J. Pickard, P. J. Hasnip, M. I. J. Probert, K. Refson and M. C. Payne, *Zeitschrift fur Kristallographie*, 2005, **220**, 567–570.

13 J. P. Perdew, A. Ruzsinszky, G. I. Csonka, O. A. Vydrov, G. E. Scuseria, L. A. Constantin, X. Zhou and K. Burke, *Phys Rev Lett*, 2008, **100**, 136406.

14 J. R. Yates, C. J. Pickard and F. Mauri, *Phys Rev B Condens Matter Mater Phys*, , DOI:10.1103/PhysRevB.76.024401.

15 A. Tkatchenko and M. Scheffler, *Phys Rev Lett*, , DOI:10.1103/PhysRevLett.102.073005.

16 H. J. Monkhorst and J. D. Pack, *Phys Rev B*, 1976, **13**, 5188–5192.

17 M. J. Frisch, G. W. Trucks, H. B. Schlegel, G. E. Scuseria, M. A. Robb, J. R. Cheeseman, G. Scalmani, V. Barone, G. A. Petersson, H. Nakatsuji, X. Li, M. Caricato, A. V Marenich, J. Bloino, B. G. Janesko, R. Gomperts, B. Mennucci, H. P. Hratchian, J. V Ortiz, A. F. Izmaylov, J. L. Sonnenberg, D. Williams-Young, F. Ding, F. Lipparini, F. Egidi, J. Goings, B. Peng, A. Petrone, T. Henderson, D. Ranasinghe, V. G. Zakrzewski, J. Gao, N. Rega, G. Zheng, W. Liang, M. Hada, M. Ehara, K. Toyota, R. Fukuda, J. Hasegawa, M. Ishida, T. Nakajima, Y. Honda, O. Kitao, H. Nakai, T. Vreven, K. Throssell, J. A. Montgomery Jr., J. E. Peralta, F. Ogliaro, M. J. Bearpark, J. J. Heyd, E. N. Brothers, K. N. Kudin, V. N. Staroverov, T. A. Keith, R. Kobayashi, J. Normand, K. Raghavachari, A. P. Rendell, J. C. Burant, S. S. Iyengar, J. Tomasi, M. Cossi, J. M. Millam, M. Klene, C. Adamo, R. Cammi, J. W. Ochterski, R. L. Martin, K. Morokuma, O. Farkas, J. B. Foresman and D. J. Fox, 2016.

18 A. Austin, N. J. Hestand, I. G. McKendry, C. Zhong, X. Zhu, M. J. Zdilla, F. C. Spano and J. M. Szarko, *J Phys Chem Lett*, 2017, **8**, 1118–1123.

19 V. Sharma, U. Puthumana, P. Karak and A. L. Koner, *J Org Chem*, 2018, **83**, 11458–11462.

20 C. Schierl, A. Niazov-Elkan, L. J. W. Shimon, Y. Feldman, B. Rybtchinski and D. M. Guldi, *Nanoscale*, 2018, **10**, 20147–20154.
21 M. Tominaga, M. Kawahata, T. Itoh and K. Yamaguchi, *Cryst Growth Des*, 2018, **18**, 37–41.
22 B. Liu, M. Böckmann, W. Jiang, N. L. Doltsinis and Z. Wang, *J Am Chem Soc*, 2020, **142**, 7092–7099.
23 G. Goretzki, E. S. Davies, S. P. Argent, J. E. Warren, A. J. Blake and N. R. Champness, *Inorg Chem*, 2009, **48**, 10264–10274.
24 Y. Zhen, H. Qian, J. Xiang, J. Qu and Z. Wang, *Org Lett*, 2009, **11**, 3084–3087.
25 P. Rajasingh, R. Cohen, E. Shirman, L. J. W. Shimon and B. Rybtchinski, *J Org Chem*, 2007, **72**, 5973–5979.
26 A. D. Hendsbee, J.-P. Sun, W. K. Law, H. Yan, I. G. Hill, D. M. Spasyuk and G. C. Welch, *Chemistry of Materials*, 2016, **28**, 7098–7109.
27 M. Burian, F. Rigodanza, N. Demitri, L. Đorđević, S. Marchesan, T. Steinhartova, I. Letofsky-Papst, I. Khalakhan, E. Mourad, S. A. Freunberger, H. Amenitsch, M. Prato and Z. Syrgiannis, *ACS Nano*, 2018, **12**, 5800–5806.
